# Availability of Stage Information From Cancer Registries Providing Data to the European Cancer Information System for Six Selected Cancers

**DOI:** 10.1002/ijc.70593

**Published:** 2026-06-15

**Authors:** Cristina Bosetti, Otto Visser, Liesbet Van Eycken, David Pettersson, Joanna Julia Bartnicka, Tadeusz Dyba, Giorgia Randi, Manuela Flego, Raquel N. Carvalho, Manola Bettio, ECIS Working Group, M. Hackl, L. Van Eycken, Z. Valerianova, M. Sekerija, I. Gregoriou, L. Dušek, M. Mägi, A. Brandhorst, J. Müller‐Nordhorn, R. Kirschner‐Schwabe, A. Nennecke, A. Schützendübel, C. Justenhoven, A. Katalinic, D. Murray, D. Camerlengo, R. Galasso, V. Ciullo, G. Sampietro, C. Gasparotti, M. Marola, M. Ferrante, F. Falcini, L. Dal Maso, M. Gambino, P. Michelozzi, L. Boni, M. Villa, V. Napolioni, A. G. Russo, A. C. Fanetti, L. Cavalieri d'Oro, R. Ortolani, G. D'Orsi, M. Fusco, P. Pinna, F. Vitale, F. Manzoni, E. Migliore, L. Bisceglia, G. Cascone, L. Di Furia, R. Cavallo, D. Piras, R. Tumino, M. Mian, G. Candela, A. Caldarella, W. Mantovani, F. Stracci, M. Castelli, M. Sbaraglia, E. Liepina, M. Mousavi, G. Smailyte, O. Visser, J. Macedo, D. Coza, V. Zadnik, A. Boone, A. Lopez De Munain Marques, M. Garrido, P. Gutiérrez Meléndez, F. Botella Quijal, R. Marcos‐Gragera, M. J. Sanchez‐Perez, E. Ramalle Gomara, M. D. Chirlaque Lopez, M. Guevara Eslava, J. Galceran, I. Curjuric, S. Erny, A. Perren, B. Uginet, M. D. Ryser, M. Von Moos, M. Maspoli, M. Mousavi, A. Bordoni, I. Konzelmann, G. Defossez, J. Diebold, S. Rohrmann, D. Bennet, D. Wyn Huws, L. May

**Affiliations:** ^1^ European Commission, Joint Research Centre (JRC) Ispra Italy; ^2^ Department of Registration Netherlands Comprehensive Cancer Organisation (IKNL) Utrecht the Netherlands; ^3^ Belgian Cancer Registry Brussels Belgium; ^4^ Engineering Ingegneria Informatica S.p.A. Rome Italy; ^5^ Piksel S.r.l Milan Italy

**Keywords:** cancer registry, Europe, neoplasms, stage, TNM

## Abstract

Cancer stage at diagnosis is crucial to guide clinical decision making and support cancer surveillance, enabling meaningful international comparisons of epidemiological cancer indicators and evaluation of cancer control policies. The collection of stage information by population‐based cancer registries (PBCRs) is therefore essential, yet a comprehensive overview of stage availability across European PBCRs has been lacking. We analyzed the availability and completeness of stage at diagnosis reported by PBCRs affiliated with the European Network of Cancer Registries (ENCR) in the 2022 European Cancer Information System (ECIS) data call. The assessment focused on six major cancers, that is, breast, cervix, colorectum, gastric, lung, and prostate. Between 1990 and 2022, 67 (78%) PBCRs from 18 countries—out of 86 registries submitting incidence data to ECIS by September 2025—provided information on stage. Sixty‐five (97%) used the Tumor‐Nodes‐Metastasis (TNM) system, although four coded a substantial proportion of cases using other staging systems. Many countries showed improvements over time in reporting TNM stage. In the period 2017–2019, in most countries more than 80% of breast, cervical, colorectal, and lung cancer cases had TNM information, while gastric and prostate cancers exceeded 70%. Nevertheless, a few countries reported TNM stage for less than half of their cases. Overall, stage at diagnosis is available for a large proportion of cancer cases in Europe, demonstrating the commitment of PBCRs to high‐quality data collection. However, important gaps persist in some countries. Continued cooperation between the ENCR and ECIS is needed to support harmonized stage reporting across European registries.

## What's New?

The lack of a standardized cancer staging framework across European cancer registries has fragmented epidemiological data, making cross‐border comparisons of cancer indicators difficult. The present study assessed stage reporting for six cancer types from 86 population‐based cancer registries contributing to the European Cancer Information System. Among registries providing stage data, most used the standardized tumor‐nodes‐metastasis (TNM) system. Analyses further show that reporting completeness improved over time, with most countries recording stage information for more than 80% of cancer cases by 2017–2019. The findings reveal progress in cancer stage data collection and identify gaps that require greater alignment across Europe.

## Graphical abstract

The lack of a standardized cancer staging framework across European cancer registries has fragmented epidemiological data, making cross‐border comparisons of cancer indicators difficult. The present study assessed stage reporting for six cancer types from 86 population‐based cancer registries contributing to the European Cancer Information System. Among registries providing stage data, most used the standardized tumor‐nodes‐metastasis (TNM) system. Analyses further show that reporting completeness improved over time, with most countries recording stage information for more than 80% of cancer cases by 2017–2019. The findings reveal progress in cancer data collection and identify gaps that require greater alignment across Europe.

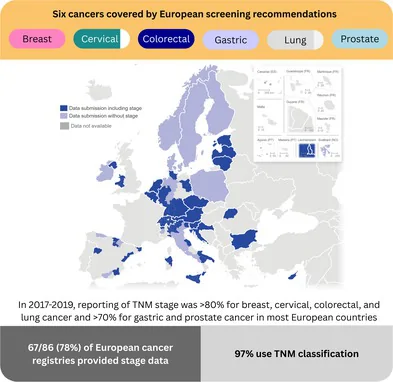

**Abbreviations**
ECIS: European Cancer Information System
ENCR: European Network of Cancer Registries
EOD: extent of disease
FIGO: International Federation of Gynecology and Obstetrics
PBCR: population‐based cancer registries
TNM: Tumor‐Nodes‐Metastasis

## 1 Introduction

Cancer stage at diagnosis is not only relevant at a clinical and patient level—since it describes the extent of disease (EOD) at the time of diagnosis and guides optimal treatment—but is also crucial for population‐level cancer surveillance [1]. Indeed, stage information allows meaningful comparisons of cancer indicators between populations by accounting for differences in the EOD at diagnosis. For example, stage‐specific survival estimates are widely used to benchmark cancer outcomes across countries and healthcare systems [2, 3]. In addition, monitoring of temporal trends in stage distribution reflects the impact of screening programs, diagnostic practices, and access to care. Consequently, the systematic collection of cancer stage is increasingly recognized as a core element of high‐quality population‐based cancer registries (PBCRs) [4, 5]. Although data on cancer stage at diagnosis are available from various European and international PBCRs [6, 7, 8, 9, 10, 11, 12, 13, 14], there are no studies providing a comprehensive overview on stage availability across European PBCRs.

We conducted an analysis to map the availability and completeness of information on stage at diagnosis provided by general PBCRs—covering all ages and all cancer types—that are affiliated with the European Network of Cancer Registries (ENCR) and submitted data within the 2022 European Cancer Information System (ECIS) data call [15, 16]. We focused on six major cancers (i.e., breast, cervical, colorectal, gastric, lung, and prostate) of specific relevance at policy level, being those for which screening programs have been (or are recommended to be) implemented in European countries and for which the appropriate registration and collection of data to monitor cancer screening performance and impact indicators is envisaged in ECIS [17]. We also focused on the Tumor‐Nodes‐Metastasis (TNM) classification system [18], which is the most frequently used staging system internationally and which describes the extent of the primary tumor (T category), the involvement of regional lymph nodes (N category), and the presence or absence of distant metastasis (M category).

## 2 Materials and Methods

### 2.1 Data Sources

The 2022 ECIS data call protocol [16] requested European PBCRs to provide:

- T, N, and M categories (clinical and pathological, whenever available);
- Information on TNM edition used [18];
- Stage already classified by the registry, with the corresponding staging system used, that is, TNM (with corresponding TNM edition), summary EOD [19], International Federation of Gynecology and Obstetrics (FIGO; for gynecological cancers) [20], or Dukes' (for colorectal cancer) staging system [16, 21].

### 2.2 Analysis

From the stage variables submitted by each CR [16], we first investigated how many PBCRs submitted any information on patients' stage at diagnosis, the first incidence year when stage information was reported by each CR, if the PBCRs provided T, N, M categories separately or only TNM stage, the TNM edition used across time‐periods by each CR, and if PBCRs provided stage coded according to staging systems other than TNM.

In addition, for each cancer we estimated the proportion of cases with (complete) information on TNM stage in the period 1990–2022. For this analysis, we considered only malignant (invasive) solid cancers with pathological confirmation; cases detected at autopsy were excluded. Cancers were classified according to the ECIS entities' definitions (see the morphology and topography codes in Table S1).

Cancer cases for whom all (clinical or pathological) T, N, M categories were available or with a valid value for TNM stage were classified as “complete”; we also considered as “complete” cases with M1 (presence of a metastasis) independently from the presence of T and N values or with a valid value for T but NX or MX (assumed to be N0/M0 [7]). Cases where information reported was insufficient to allow TNM stage to be calculated—that is, having only one or two of (clinical and/or pathological) T, N, M categories (except when M = 1), having inconsistent clinical and/or pathological T, N, M combinations (as TXN0M0), or not having the TNM edition—were classified as “unknown” [16]. Cases for whom no information on TNM stage was reported—that is, missing all clinical and pathological T, N, M categories and without TNM stage—were classified as “missing”. For cervical cancer, information on stage coded using both TNM and FIGO staging systems was considered, as there is a correspondence between the two systems.

Results were summarized at a country level, except for Romania, Portugal, and the UK, since in these countries only a few registries—covering a very low proportion of the population—contributed with data. For countries with regional CRs with incomplete national coverage—Germany: 7 of 15 active registries, 69% of the population; Italy: 38 of 39, 83%; Spain: 11 of 13, 23%; Switzerland: 13 of 13, 97%—results were obtained by pooling registry‐level data within the country.

An overall estimate of stage availability for all countries combined was also provided. This analysis focused on the period from 1990 on; due to the limited number of cases in some PBCRs and to ensure stable results, we pooled data over three‐year periods and grouped them as follows: 1990–92, 1993–95, …., 2017–19, and 2020–22 (or latest data available).

All data analyses were performed using the SAS software version 9.4 (SAS Institute Inc., Cary, NC, USA). Figures were prepared using the software R version 4.5.0 (R Development Core Team, 2025); the map was done using the QGIS Geographic Information System version 3.34 (QGIS Development Team, 2023).

## 3 Results

We considered data from 86 general PBCRs from 20 European countries, which were submitted to ECIS and validated by September 2025 (Figure S1). Of these, 67 PBCRs (78%) from 18 countries provided data on stage at diagnosis for at least one of the six cancers. Information on stage was provided from the 1980's for Austria, the Netherlands, Slovenia, and a few PBCRs from Switzerland, Italy, and Spain, from the early 1990's in Bulgaria, Czechia, Estonia, Germany, Lithuania, and the UK—Northern Ireland, and UK—Wales, since the early 2000's in Belgium and Latvia, and from the early 2010's in Liechtenstein, Lithuania, and Romania—Cluj.

Of the 67 PBCRs submitting stage, 65 PBCRs (97%) from 16 countries provided information according to the TNM classification system (Table S2). Of these, 62 (95%) provided information on single (clinical and/or pathological) T, N, M categories for most cancer cases. Cyprus and Croatia provided only stage coded using the EOD system. Moreover, a few CRs (*N* = 4) coded a substantial proportion of cancers using only the EOD staging system (Sassari, Italy 18%; Estonia 26%; Basque Country, Spain 57%; and Slovenia 70%), while a few others (*N* = 9) used other staging systems in a limited proportion of cases. Over the entire period considered (1990–2022), 3.4% of breast cancer cases were classified using EOD; 7.8% of cervical cancer cases were classified using EOD (4.4%) or FIGO (2.4%); 4.8% of colorectal cancer cases were classified using EOD (3.8%) or Dukes' (1.0%); 7.2% of all gastric cancer cases were classified using EOD; 5.0% of lung cancer cases were classified using EOD; and 4.6% of prostate cancer cases were classified using EOD.

The TNM editions used to classify stage in each PBCR by time‐period are given in Table S3. Cancer cases were coded according to first to third TNM editions in only a few Swiss PBCRs, while from the mid 2000s/early 2010s almost all PBCRs used the sixth to eighth TMN editions.

**FIGURE 1:**
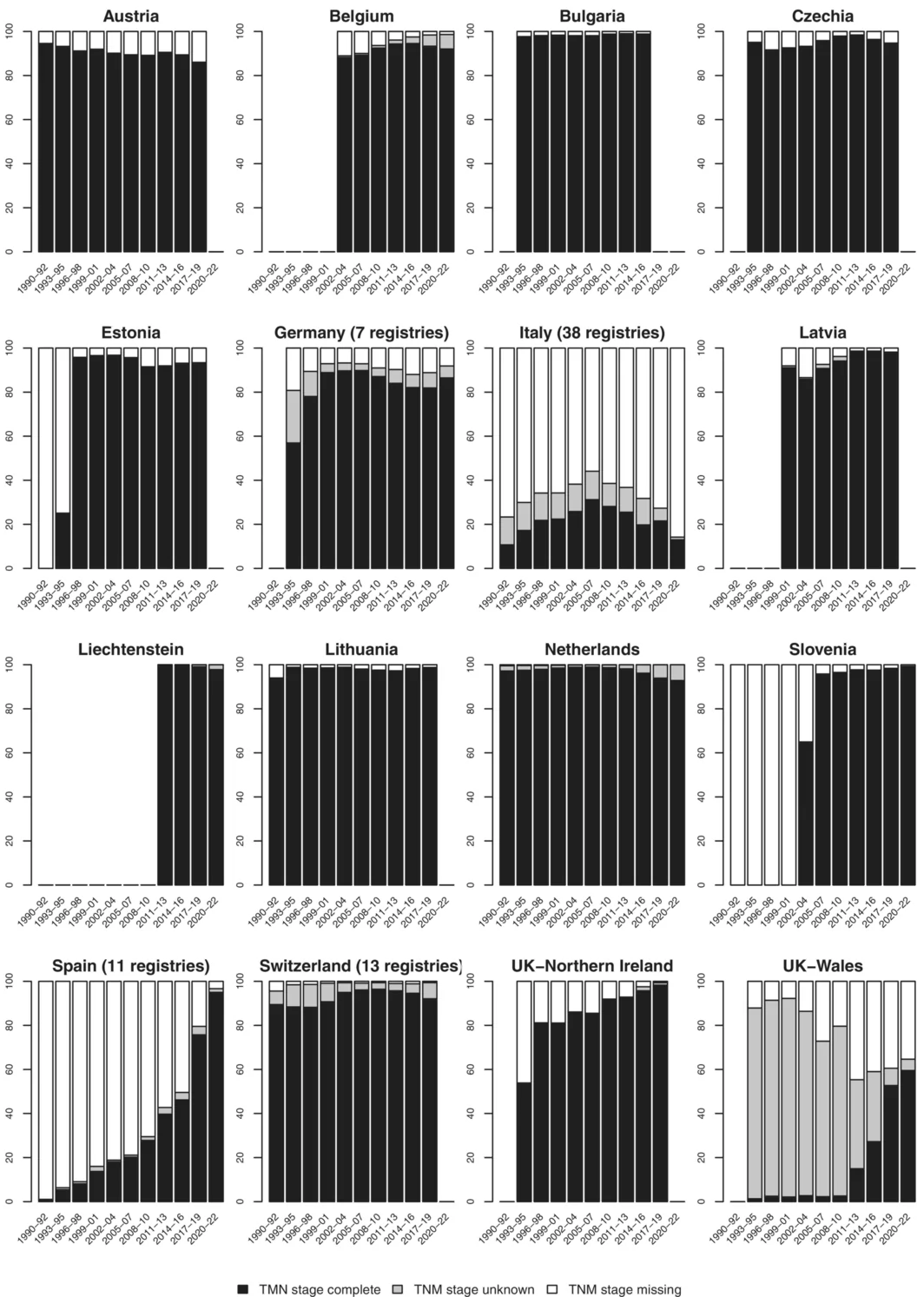
TNM stage availability (%) by country across time‐periods—Breast cancer.

**FIGURE 2:**
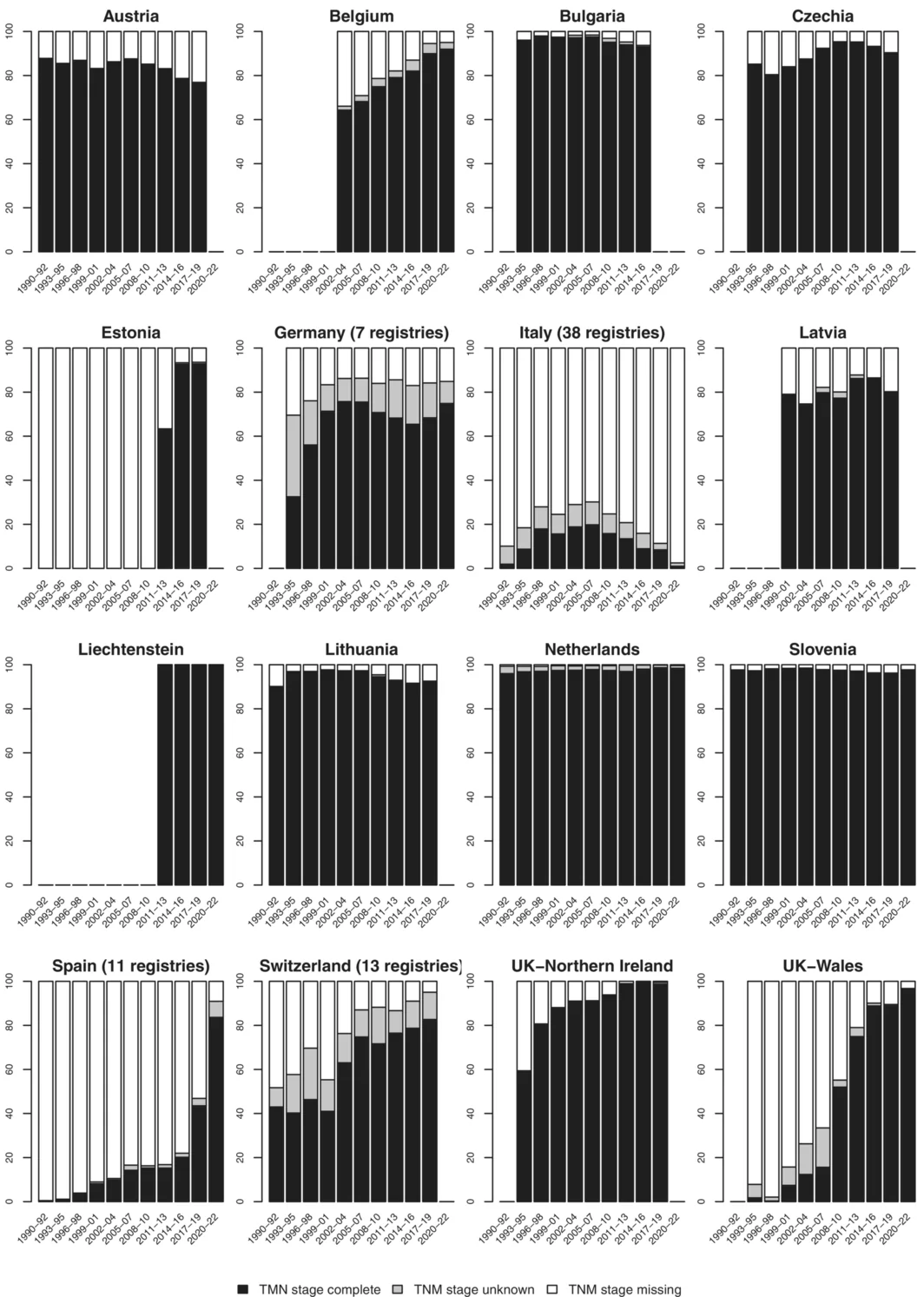
TNM stage availability (%) by country across time‐periods—Cervical cancer.

**FIGURE 3:**
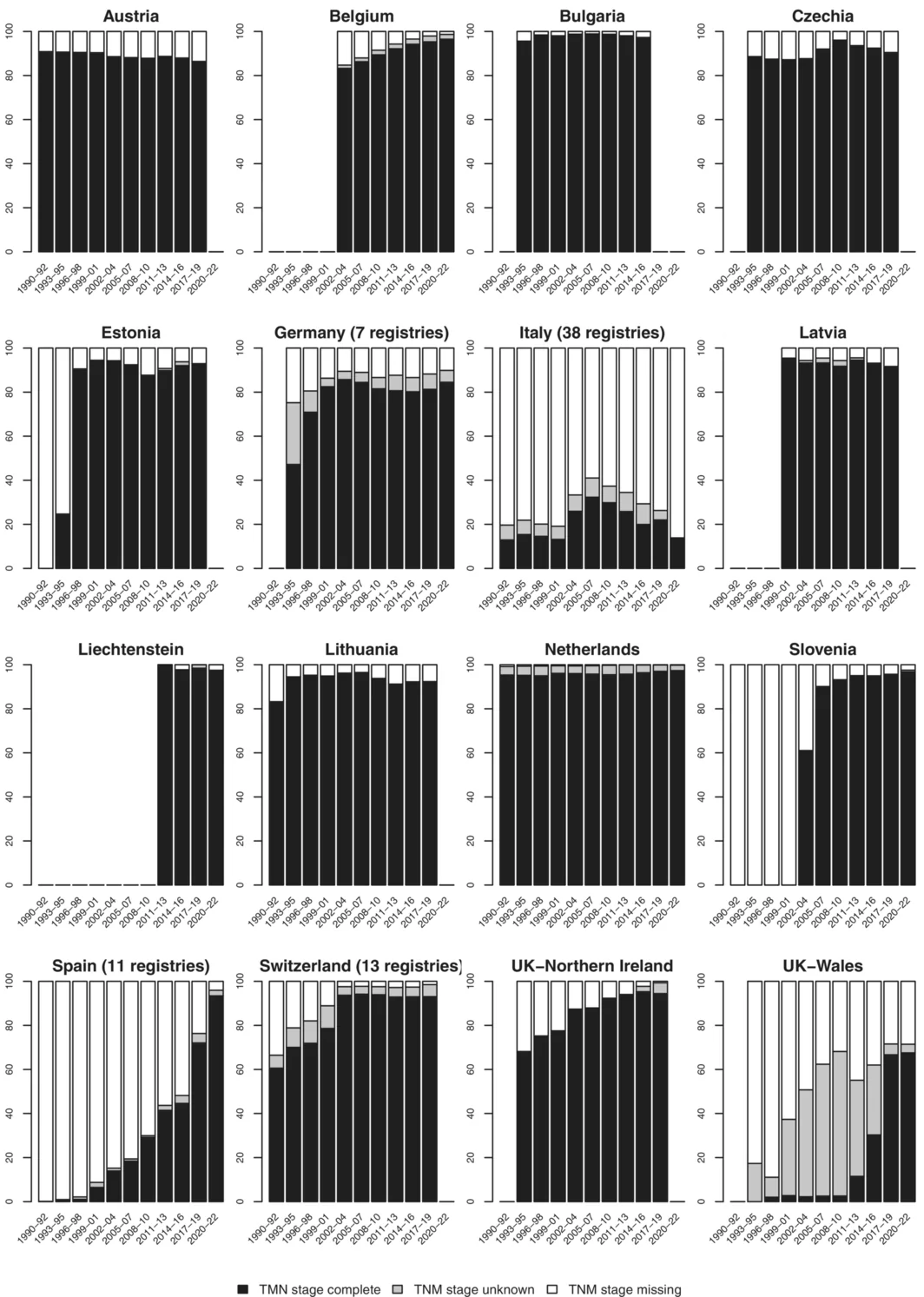
TNM stage availability (%) by country across time‐periods—Colorectal cancer.

**FIGURE 4:**
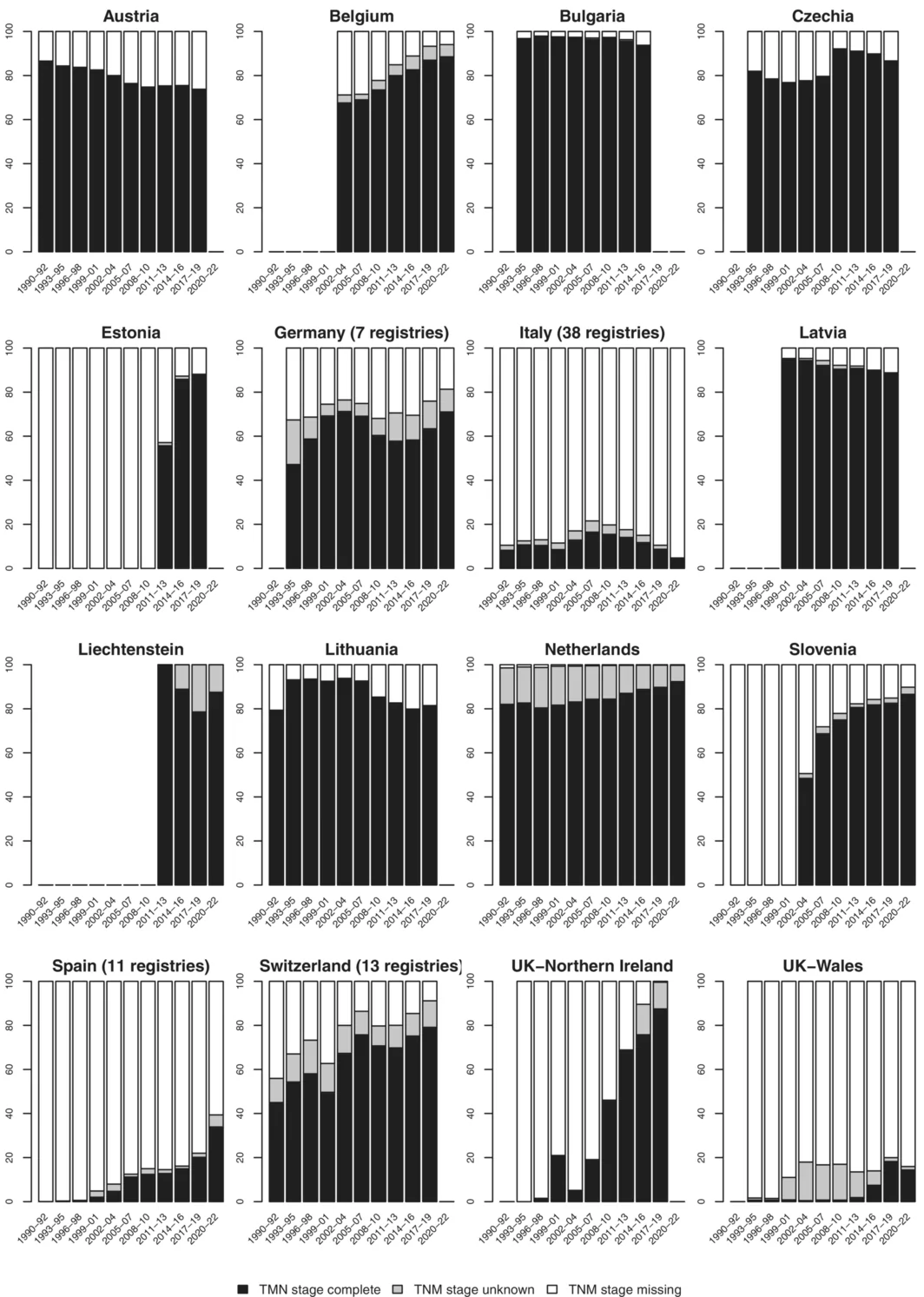
TNM stage availability (%) by country across time‐periods—Gastric cancer.

**FIGURE 5:**
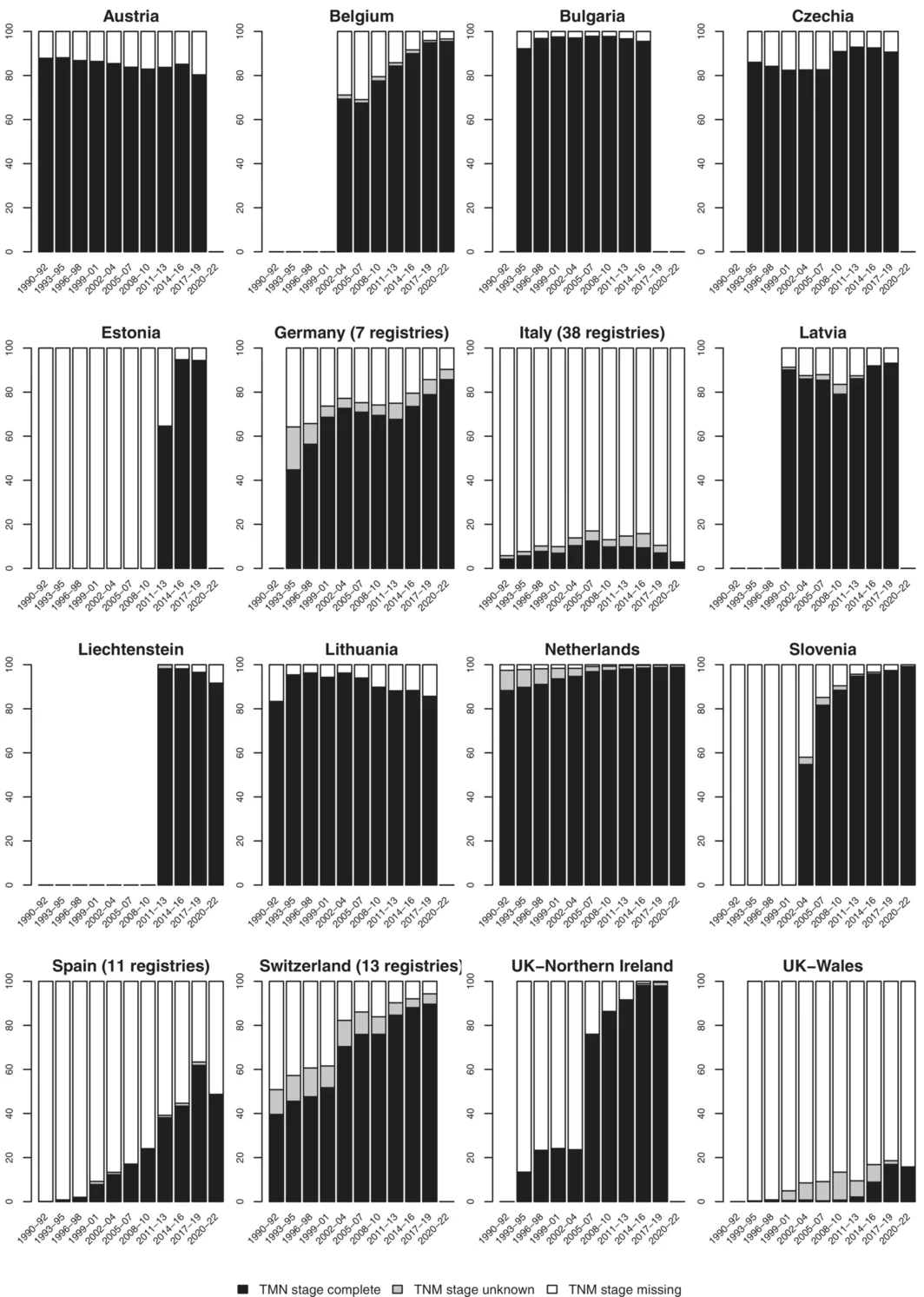
TNM stage availability (%) by country across time‐periods—Lung cancer.

**FIGURE 6:**
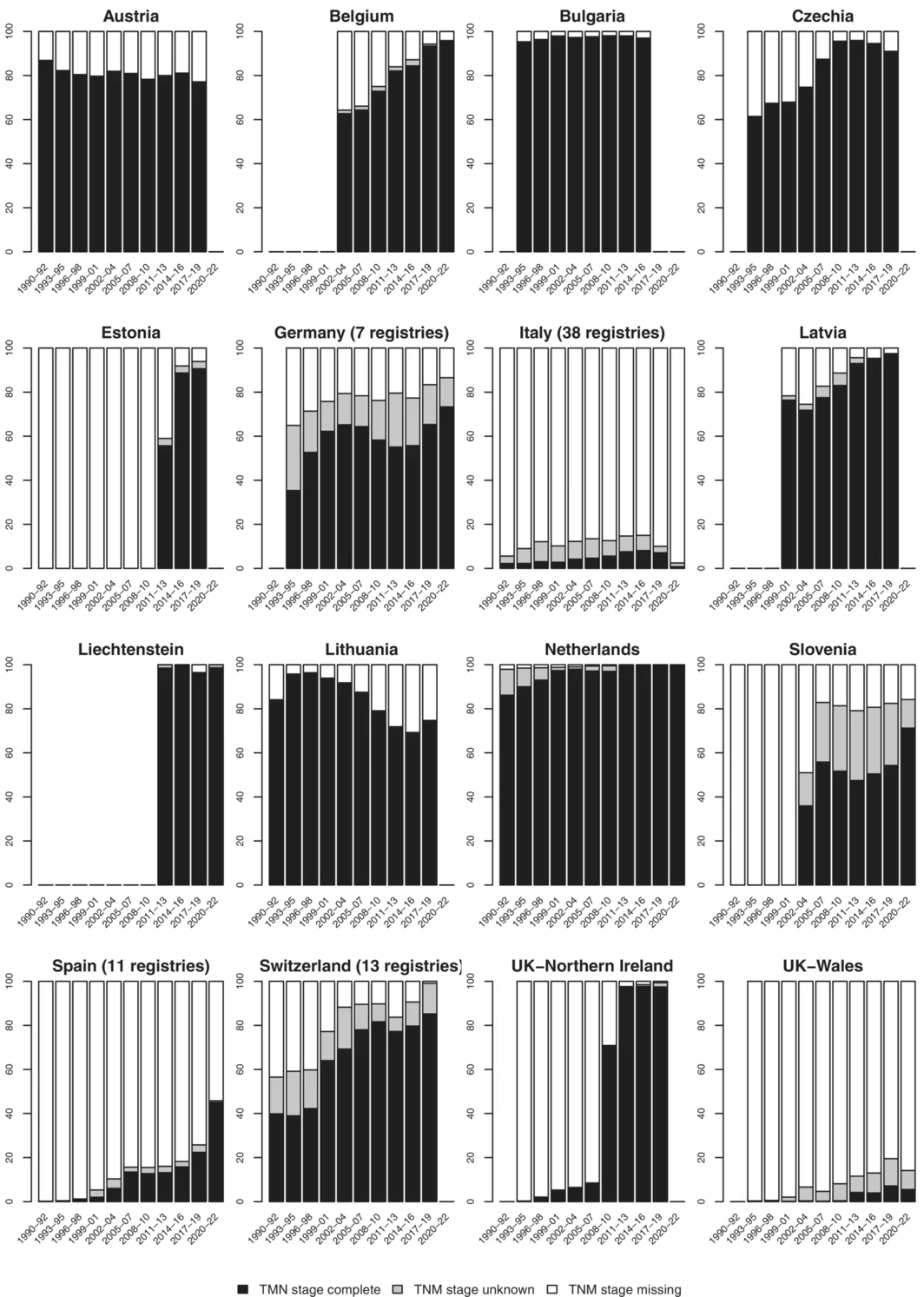
TNM stage availability (%) by country across time‐periods—Prostate cancer.

Figures 1, 2, 3, 4, 5, 6 show the proportion of cancer cases for whom TNM stage at diagnosis was submitted to ECIS for the six cancers considered in 16 European countries (excluding Cyprus and Croatia since they did not report TNM stage) and across the time‐periods considered.

Over the period 1990–2022, 2,787,229 breast, 239,119 cervical, 2,543,740 colorectal, 699,062 gastric, 2,353,747 lung, and 2,328,353 prostate cancers were considered; of these, 4.9%, 5.0%, 2.7%, 14.0%, 20.7%, and 8.2% without pathological confirmation were excluded, respectively. Total numbers of cancer cases and percentages of cases with complete, unknown or missing information on TNM stage for the six cancers considered—by countries and time‐period—are given in Tables S4–S9. Apart from countries with national coverage, the number of cancer cases may vary across time‐periods, depending on how many regional PBCRs contributed data during these periods.

### 3.1 Breast Cancer

Complete TNM stage information was reported for a high proportion of breast cancer cases in most European countries over the period considered, except Italy and Spain (Figure 1 and Table S4). In Spain, reporting of TNM stage information steadily increased across time periods.

For the period 2017–19—that is, the most recent time‐period with stage information from most PBCRs—complete TNM stage information was reported for over 90% of breast cancer cases in Belgium, Bulgaria (in 2014–16, last period available), Czechia, Estonia, Latvia, Liechtenstein, Lithuania, the Netherlands, Slovenia, UK—Northern Ireland and between 80% and 90% of cases in Austria, Germany, and Switzerland. Proportions were lower in Spain (76%), the UK—Wales (53%), and Italy (22%). In all countries combined, 70% of breast cancer cases had a complete TNM classification.

### 3.2 Cervical Cancer

The proportion of cervical cancer cases with complete information reported for TNM stage was high in most European countries over the period considered, and increased over time in Belgium, Estonia, Spain, and Switzerland. Only in Italy did it remain low for most time‐periods (Figure 2 and Table S5).

In 2017–19, complete TNM stage was reported for at least 90% of cervical cancer cases in Belgium, Bulgaria (in 2014–16), Czechia, Estonia, Lithuania, the Netherlands, Slovenia, and the UK—Northern Ireland, and between 70% and 90% of cases in Austria, Latvia, Switzerland, and UK—Wales. Proportions were 68% in Germany, 44% in Spain, and 8% in Italy. In all countries combined, 64% of cervical cancer cases had a complete TNM classification.

### 3.3 Colorectal Cancer

The proportion of colorectal cancer cases with complete TNM stage reporting was high in many European countries over the period considered, and it increased across time‐periods in Belgium, Germany, Slovenia, Spain, and Switzerland (Figure 3 and Table S6). TNM stage reporting was missing/unknown for a large proportion of cases in Italy.

In 2017–19, TNM stage information was reported for over 90% of colorectal cancer cases in Belgium, Bulgaria (in 2014–16, last period available), Czechia, Estonia, Latvia, Liechtenstein, Lithuania, the Netherlands, Slovenia, Switzerland, and the UK—Northern Ireland and for 80% to 90% of cases in Austria and Germany. Proportions were 72% in Spain, 67% in the UK—Wales, and 22% in Italy. In all countries combined, 71% of colorectal cancer cases had a complete TNM classification.

### 3.4 Gastric Cancer

Complete information on TNM stage was reported for a high proportion of gastric cancer cases in Bulgaria, Czechia, Liechtenstein, and the Netherlands over the time‐period considered and for an increasing proportion of cases across time‐periods in other European countries such as Belgium, Estonia, Germany, Slovenia, Spain, and Switzerland (Figure 4 and Table S7). Reporting on TNM stage remained low in Italy and Spain.

In the period 2017–19, over 80% of gastric cancer cases had a complete TNM classification in Belgium, Bulgaria (in 2014–16, last period available), Czechia, Estonia, Latvia, Liechtenstein, Lithuania, the Netherlands, Slovenia, and UK—Northern Ireland and between 70% and 80% in Austria and Switzerland. Complete information on TNM stage was lower in Germany (63%), Spain (20%), UK—Wales (39%), and Italy (9%). In all countries combined, 52% of gastric cancer cases had a complete TNM classification.

### 3.5 Lung Cancer

Complete TNM stage information was reported for a high proportion of lung cancer cases in Austria, Bulgaria, Czechia, Liechtenstein, Lithuania, and the Netherlands over the period considered, while it increased across time‐periods in Belgium, Estonia, Germany, Slovenia, Spain, and Switzerland (Figure 5 and Table S8). TNM stage information was low for most time‐periods in Italy and Spain.

In 2017–19, complete TNM stage information was reported for over 90% of lung cancer cases in Belgium, Bulgaria (in 2014–16), Estonia, Latvia, Liechtenstein, the Netherlands, UK‐ Northern Ireland, and Slovenia, and 80% to 90% of cases in Austria, Czechia, Germany, Lithuania, and Switzerland. The proportion was 62% in Spain, 17% in UK‐Wales, and 7% in Italy. In all countries combined, 68% of lung cancer cases had a complete TNM classification.

### 3.6 Prostate Cancer

The proportion of prostate cancer cases with complete TNM stage information reported was high over the period considered in Austria, Bulgaria, Liechtenstein, and the Netherlands; it increased across time‐periods in Belgium, Czechia, Estonia, Germany, Latvia, Spain, Slovenia, and Switzerland. It remained low in Italy and Spain but decreased in Lithuania (Figure 6 and Table S9).

In 2017–19, TNM stage information was provided for over 90% of prostate cancer cases in Belgium, Bulgaria (2014–16), Czechia, Estonia, Latvia, Liechtenstein, the Netherlands, and UK‐Northern Ireland for between 80% and 90% of cases in Switzerland, and for between 70% and 80% of cases in Austria and Lithuania. Lower completeness was found for the following countries: 65% in Germany, 54% in Slovenia, 22% in Spain, and 7% in Italy and UK‐Wales. In all countries combined, 60% of prostate cancer cases had a complete TNM classification.

## 4 Discussion

Our analyses showed that 78% of PBCRs which sent data for the 2022 ECIS data call [15, 16] provided information on cancer stage at diagnosis. All but two PBCRs coded their cancer cases using the TNM staging system, and approximately 95% of them provided information on single (clinical and/or pathological) T, N, M categories, which will allow to calculate TNM stage in a harmonized manner for better international comparability. TNM stage information was provided by PBCRs using different TNM editions—first through eighth—across time‐periods [18]. Changes between TNM editions may affect stage definitions, although the magnitude of these changes varies by cancer site; for example, substantial revisions have occurred for lung cancer staging, whereas stage groupings for colorectal cancer have remained relatively stable across editions [22]. Heterogeneity in classification systems used and limitations in the comparability across PBCRs have indeed been frequently reported in the literature [2, 6, 8, 23], this also highlighting the importance of officially translated TNM editions into European languages, as well as the adoption of the most recent TNM edition to ensure better comparability of stage data across registries.

We found high variability in the availability and completeness of the provided TNM stage information across time‐periods, countries, and cancers, as also reported in a recent web‐based survey [13]. With reference to time‐periods, The Netherlands and Bulgaria provided complete TNM stage information for a high proportion of cancer cases over the entire period considered, while in many other European countries—namely Belgium, Estonia, Slovenia, Spain and Switzerland—there was a steady increase in the proportion of cancer cases with complete TNM stage information over time, partly explained by the increased digitalization and better accessibility to clinical reports. A web‐based survey on 141 PBCRs −85 from high income countries, 48 from Europe—found that the percentages of stage recording were over 90% in high income countries, with higher percentages compared to those reported in the past [10].

With regards to differences between countries, over the most recent time‐period (2017–19) the proportion of cancer cases with information on TNM stage was higher than 80% for breast, cervical, colorectal, and lung cancer and over 70% for gastric and prostate cancer in several European countries. However, TNM stage reporting was lower (< 50%) in Italy and Spain for most cancers. Moreover, there was a substantial variation in the completeness of stage information across regional registries within a country; even within Italy and Spain there were a few registries reporting high stage coverage for the cancers considered. TNM stage information reporting was low in Germany for cervical, gastric, and prostate cancer and in Slovenia for prostate cancer. The low proportion of cancer cases with TNM stage information in a few countries (e.g., Slovenia for prostate cancer) might be partly because these PBCRs coded a quite large proportion of cancer cases using staging systems other than TNM, particularly in the most remote years.

Our study does not fully capture the availability of TNM stage information across all European PBCR, since we considered only PBCRs providing stage information to ECIS by September 2025. There might be a few additional PBCRs which have provided incidence data to ECIS having also registered stage information—as for example Ireland, Caserta, Lazio, Tuscany, and Veneto (Italy) and Asturias (Spain)—but which have not yet sent stage data to ECIS, possibly due to the lack of validation or potential inaccuracies in stage information. Moreover, additional ENCR‐affiliated PBCRs which only submitted aggregated data—such as Nordic ones—and other PBCRs which had not yet contributed incidence data to ECIS were not considered in our analysis. Furthermore, except for countries with a national cancer registration, estimates of stage availability may not completely reflect the whole country, since registries included in our analysis covered only a variable percentage of the country population.

## 5 Conclusion

Notwithstanding the limitations highlighted above, our study contributes to a better understanding of the availability and completeness of information on cancer stage at diagnosis provided by PBCRs in Europe. While many countries report stage for a high proportion of cancer cases—demonstrating the commitment of PBCRs to high‐quality data collections—important gaps persist in some countries, indicating considerable room for improvement. Moreover, a couple of registries have not yet registered stage using the TNM system. Accurate recording TNM is complex, requiring trained staff, access to pathology reports, and close collaboration with oncology departments, all of which can be limited by financial and logistical constraints. Additional challenges include incomplete communication between physicians, inconsistent use of TNM in clinical practice, and lack of mandatory reporting. Solutions such as automation and Artificial Intelligence‐driven extraction of TNM from medical records could improve stage completeness and quality. Strengthening collaboration between clinical oncologists and PBCRs and promoting best practices in TNM reporting can further enhance the availability and quality of stage information. Continued cooperation between the ENCR and ECIS is needed to identify and address these challenges and to support harmonized stage reporting across European registries. Ultimately, the provision of TNM stage at diagnosis by European PBCRs will allow the inclusion of stage information in the ECIS web‐application (www.ecis.jrc.ec.europa.eu), to support clinical, epidemiological, and policy decision‐making.

## Author Contributions

**Cristina Bosetti:** conceptualization, methodology, formal analysis, writing – original draft, writing – review and editing. **Otto Visser:** writing – original draft, writing – review and editing, methodology. **Liesbet Van Eycken:** writing – original draft, writing – review and editing, methodology. **David Pettersson:** methodology, writing – review and editing, conceptualization. **Joanna Julia Bartnicka:** data curation, writing – review and editing. **Tadeusz Dyba:** data curation, writing – review and editing. **Giorgia Randi:** data curation, writing – review and editing. **Manuela Flego:** data curation, writing – review and editing. **Raquel N. Carvalho:** project administration, writing – review and editing. **Manola Bettio:** conceptualization, writing – review and editing, project administration.

## Conflicts of Interest

The authors declare no conflicts of interest.

## Supporting information


**Table S1:** Cancer entities' topography and morphology codes.
**Table S2:** Information on cancer stage at diagnosis available by cancer registry.
**Table S3:** TNM edition adopted by cancer registry across calendar period.
**Table S4:** Breast cancer cases with information on TNM stage by country across calendar periods.
**Table S5:** Cervical cancer cases with information on TNM stage by country across calendar periods.
**Table S6:** Colorectal cancer cases with information on TNM stage by country across calendar periods.
**Table S7:** Gastric cancer cases with information on TNM stage by country across calendar periods.
**Table S8:** Lung cancer cases with information on TNM stage by country across calendar periods.
**Table S9:** Prostate cancer cases with information on TNM stage by country across calendar periods.
**Figure S1:** General European population‐based cancer registries providing information on cancer stage at diagnosis, September 2025.

## Data Availability

The data supporting this study's findings are held by European Cancer Registries and were used under a specific data‐sharing agreement with the European Commission Joint Research Centre. The anonymized dataset and further information that support the findings of this study are available from the corresponding author upon reasonable request. All source code is publicly available on GitHub https://github.com/crisbosetti‐art/Stage‐availability.git.
