# Supplementary material for: Availability of Stage Information From Cancer Registries Providing Data to the European Cancer Information System for Six Selected Cancers
Source: Int J Cancer. 2026 Jun 15;159(10):2540–51. doi: 10.1002/ijc.70593 (PMC13595485; doi:10.1002/ijc.70593)

SUPPLEMENTARY MATERIAL

Manuscript Title

Availability of stage information from cancer registries providing data to the European cancer information system for six selected cancers

Author list

Cristina Bosetti, Otto Visser, Liesbet Van Eycken, David Pettersson, Joanna Julia Bartnicka, Tadeusz Dyba, Giorgia Randi, Manuela Flego, Raquel N. Carvalho, ECIS Working Group

Table of contents

Supplementary Table 1. Cancer entities' topography and morphology codes.

Supplementary Table 2. Information on cancer stage at diagnosis available by cancer registry.

Supplementary Table 3. TNM edition adopted by cancer registry across calendar periods.

Supplementary Table 4. Breast cancer cases with available information on TNM stage by country across calendar periods.

Supplementary Table 5. Cervical cancer cases with available information on TNM stage by country across calendar periods.

Supplementary Table 6. Colorectal cancer cases with available information on TNM stage by country across calendar periods.

Supplementary Table 7. Gastric cancer cases with available information on TNM stage by country across calendar periods.

Supplementary Table 8. Lung cancer cases with available information on TNM stage by country across calendar periods.

Supplementary Table 9. Prostate cancer cases with available information on TNM stage by country across calendar periods.

Supplementary Figure 1. General European population-based cancer registries providing information on cancer stage at diagnosis, September 2025.

Supplementary Table 1. Cancer entities' topography and morphology codes.

Cancer	Topography (ICD-0)	Morphology
Carcinoma of the breast	C50	8000, 8001, 8002, 8003, 8004, 8005, 8010, 8011, 8012, 8013, 8014, 8015, 8020, 8021, 8022, 8030, 8031, 8032, 8033, 8034, 8035, 8041, 8042, 8043, 8044, 8045, 8046, 8050, 8051, 8052, 8054, 8070, 8071, 8072, 8073, 8074, 8075, 8076, 8078, 8082, 8083, 8084, 8085, 8086, 8090, 8091, 8092, 8093, 8094, 8095, 8097, 8098, 8100, 8102, 8110, 8120, 8121, 8122, 8123, 8124, 8130, 8131, 8140, 8141, 8142, 8143, 8144, 8145, 8147, 8150, 8151, 8152, 8153, 8154, 8155, 8156, 8158, 8160, 8161, 8162, 8163, 8170, 8171, 8172, 8173, 8174, 8175, 8180, 8190, 8200, 8201, 8210, 8211, 8213, 8214, 8215, 8220, 8221, 8230, 8231, 8240, 8241, 8242, 8243, 8244, 8245, 8246, 8247, 8249, 8250, 8251, 8252, 8253, 8254, 8255, 8256, 8257, 8260, 8261, 8262, 8263, 8265, 8270, 8272, 8273, 8280, 8281, 8290, 8300, 8310, 8311, 8312, 8313, 8314, 8315, 8316, 8317, 8318, 8319, 8320, 8322, 8323, 8330, 8331, 8332, 8333, 8335, 8337, 8339, 8340, 8341, 8342, 8343, 8344, 8345, 8346, 8347, 8350, 8370, 8380, 8381, 8382, 8383, 8384, 8390, 8400, 8401, 8402, 8403, 8406, 8407, 8408, 8409, 8410, 8413, 8420, 8430, 8440, 8441, 8450, 8452, 8453, 8460, 8461, 8470, 8471, 8474, 8480, 8481, 8482, 8490, 8500, 8501, 8502, 8503, 8504, 8507, 8509, 8510, 8512, 8513, 8514, 8520, 8521, 8522, 8523, 8524, 8525, 8530, 8540, 8541, 8542, 8543, 8550, 8551, 8552, 8560, 8562, 8570, 8571, 8572, 8573, 8574, 8575, 8576, 8940, 8941, 8950, 8980, 8981, 8982, 8983, 9110/3
Carcinoma of the cervix uteri	C53	8000, 8001, 8002, 8003, 8004, 8005, 8010, 8011, 8012, 8013, 8014, 8015, 8020, 8021, 8022, 8030, 8031, 8032, 8033, 8034, 8035, 8041, 8042, 8043, 8044, 8045, 8046, 8050, 8051, 8052, 8054, 8070, 8071, 8072, 8073, 8074, 8075, 8076, 8078, 8082, 8083, 8084, 8085, 8086, 8090, 8091, 8092, 8093, 8094, 8095, 8097, 8098, 8100, 8102, 8110, 8120, 8121, 8122, 8123, 8124, 8130, 8131, 8140, 8141, 8142, 8143, 8144, 8145, 8147, 8150, 8151, 8152, 8153, 8154, 8155, 8156, 8158, 8160, 8161, 8162, 8163, 8170, 8171, 8172, 8173, 8174, 8175, 8180, 8190, 8200, 8201, 8210, 8211, 8213, 8214, 8215, 8220, 8221, 8230, 8231, 8240, 8241, 8242, 8243, 8244, 8245, 8246, 8247, 8249, 8250, 8251, 8252, 8253, 8254, 8255, 8256, 8257, 8260, 8261, 8262, 8263, 8265, 8270, 8272, 8273, 8280, 8281, 8290, 8300, 8310, 8311, 8312, 8313, 8314, 8315, 8316, 8317, 8318, 8319, 8320, 8322, 8323, 8330, 8331, 8332, 8333, 8335, 8337, 8339, 8340, 8341, 8342, 8343, 8344, 8345, 8346, 8347, 8350, 8370, 8380, 8381, 8382, 8383, 8384, 8390, 8400, 8401, 8402, 8403, 8406, 8407, 8408, 8409, 8410, 8413, 8420, 8430, 8440, 8441, 8450, 8452, 8453, 8460, 8461, 8470, 8471, 8474, 8480, 8481, 8482, 8490, 8500, 8501, 8502, 8503, 8504, 8507, 8509, 8510, 8512, 8513, 8514, 8520, 8521, 8523, 8524, 8525, 8530, 8540, 8541, 8542, 8543, 8550, 8551, 8552, 8560, 8562, 8570, 8571, 8572, 8573, 8574, 8575, 8576, 8940, 8941, 8950, 8980, 8981, 8982, 8983, 9110/3
Carcinoma of the colon, rectum and anus	C18-C21	8000, 8001, 8002, 8003, 8004, 8005, 8010, 8011, 8012, 8013, 8014, 8015, 8020, 8021, 8022, 8030, 8031, 8032, 8033, 8034, 8035, 8041, 8042, 8043, 8044, 8045, 8046, 8050, 8051, 8052, 8054, 8070, 8071, 8072, 8073, 8074, 8075, 8076, 8078, 8082, 8083, 8084, 8085, 8086, 8090, 8091, 8092, 8093, 8094, 8095, 8097, 8098, 8100, 8102, 8110, 8120, 8121, 8122, 8123, 8124,

		8130, 8131, 8140, 8141, 8142, 8143, 8144, 8145, 8147, 8154, 8160, 8161, 8162, 8163, 8170, 8171, 8172, 8173, 8174, 8175, 8180, 8190, 8200, 8201, 8210, 8211, 8213, 8214, 8215, 8220, 8221, 8230, 8231, 8243, 8244, 8245, 8246/3, 8246/4, 8246/9, 8250, 8251, 8252, 8253, 8254, 8255, 8256, 8257, 8260, 8261, 8262, 8263, 8265, 8270, 8272, 8273, 8280, 8281, 8290, 8300, 8310, 8311, 8312, 8313, 8314, 8315, 8316, 8317, 8318, 8319, 8320, 8322, 8323, 8330, 8331, 8332, 8333, 8335, 8337, 8339, 8340, 8341, 8342, 8343, 8344, 8345, 8346, 8347, 8350, 8370, 8380, 8381, 8382, 8383, 8384, 8390, 8400, 8401, 8402, 8403, 8406, 8407, 8408, 8409, 8410, 8413, 8420, 8430, 8440, 8441, 8450, 8452, 8453, 8460, 8461, 8470, 8471, 8474, 8480, 8481, 8482, 8490, 8500, 8501, 8502, 8503, 8504, 8507, 8509, 8510, 8512, 8513, 8514, 8520, 8521, 8523, 8524, 8525, 8530, 8540, 8541, 8542, 8543, 8550, 8551, 8552, 8560, 8562, 8570, 8571, 8572, 8573, 8574, 8575, 8576, 8940, 8941, 8950, 8980, 8981, 8982, 8983/3
Gastric carcinoma	C19	8000, 8001, 8002, 8003, 8004, 8005, 8010, 8011, 8012, 8013, 8014, 8015, 8020, 8021, 8022, 8030, 8031, 8032, 8033, 8034, 8035, 8041, 8042, 8043, 8044, 8045, 8046, 8050, 8051, 8052, 8054, 8070, 8071, 8072, 8073, 8074, 8075, 8076, 8078, 8082, 8083, 8084, 8085, 8086, 8090, 8091, 8092, 8093, 8094, 8095, 8097, 8098, 8100, 8102, 8110, 8120, 8121, 8122, 8123, 8124, 8130, 8131, 8140, 8141, 8142, 8143, 8144, 8145, 8147, 8154, 8160, 8161, 8162, 8163, 8170, 8171, 8172, 8173, 8174, 8175, 8180, 8190, 8200, 8201, 8210, 8211, 8213, 8214, 8215, 8220, 8221, 8230, 8231, 8243, 8244, 8245, 8246/3, 8246/4, 8246/9, 8250, 8251, 8252, 8253, 8254, 8255, 8256, 8257, 8260, 8261, 8262, 8263, 8265, 8270, 8272, 8273, 8280, 8281, 8290, 8300, 8310, 8311, 8312, 8313, 8314, 8315, 8316, 8317, 8318, 8319, 8320, 8322, 8323, 8330, 8331, 8332, 8333, 8335, 8337, 8339, 8340, 8341, 8342, 8343, 8344, 8345, 8346, 8347, 8350, 8370, 8380, 8381, 8382, 8383, 8384, 8390, 8400, 8401, 8402, 8403, 8406, 8407, 8408, 8409, 8410, 8413, 8420, 8430, 8440, 8441, 8450, 8452, 8453, 8460, 8461, 8470, 8471, 8474, 8480, 8481, 8482, 8490, 8500, 8501, 8502, 8503, 8504, 8507, 8509, 8510, 8512, 8513, 8514, 8520, 8521, 8523, 8524, 8525, 8530, 8540, 8541, 8542, 8543, 8550, 8551, 8552, 8560, 8562, 8570, 8571, 8572, 8573, 8574, 8575, 8576, 8940, 8941, 8950, 8980, 8981, 8982, 8983/3
Carcinoma of the bronchus and lung	C34	8000, 8001, 8002, 8003, 8004, 8005, 8010, 8011, 8012, 8013, 8014, 8015, 8020, 8021, 8022, 8030, 8031, 8032, 8033, 8034, 8035, 8041, 8042, 8043, 8044, 8045, 8046, 8050, 8051, 8052, 8054, 8070, 8071, 8072, 8073, 8074, 8075, 8076, 8078, 8082, 8083, 8084, 8085, 8086, 8090, 8091, 8092, 8093, 8094, 8095, 8097, 8098, 8100, 8102, 8110, 8120, 8121, 8122, 8123, 8124, 8130, 8131, 8140, 8141, 8142, 8143, 8144, 8145, 8147, 8150, 8151, 8152, 8153, 8154, 8155, 8156, 8158, 8160, 8161, 8162, 8163, 8170, 8171, 8172, 8173, 8174, 8175, 8180, 8190, 8200, 8201, 8210, 8211, 8213, 8214, 8215, 8220, 8221, 8230, 8231, 8240, 8241, 8242, 8243, 8244, 8245, 8246, 8247, 8249, 8250, 8251, 8252, 8253, 8254, 8255, 8256, 8257, 8260, 8261, 8262, 8263, 8265, 8270, 8272, 8273, 8280, 8281, 8290, 8300, 8310, 8311, 8312, 8313, 8314, 8315, 8316, 8317, 8318, 8319, 8320, 8322, 8323, 8330, 8331, 8332, 8333, 8335, 8337, 8339, 8340, 8341, 8342, 8343, 8344, 8345, 8346, 8347, 8350, 8370, 8380, 8381, 8382, 8383, 8384, 8390, 8400, 8401, 8402, 8403, 8406,

		8407, 8408, 8409, 8410, 8413, 8420, 8430, 8440, 8441, 8450, 8452, 8453, 8460, 8461, 8470, 8471, 8474, 8480, 8481, 8482, 8490, 8500, 8501, 8502, 8503, 8504, 8507, 8509, 8510, 8512, 8513, 8514, 8520, 8521, 8523, 8524, 8525, 8530, 8540, 8541, 8542, 8543, 8550, 8551, 8552, 8560, 8562, 8570, 8571, 8572, 8573, 8574, 8575, 8576, 8940, 8941, 8950, 8980, 8981, 8982, 8983/3
Carcinoma of the prostate	C61	8000, 8001, 8002, 8003, 8004, 8005, 8010, 8011, 8012, 8013, 8014, 8015, 8020, 8021, 8022, 8030, 8031, 8032, 8033, 8034, 8035, 8041, 8042, 8043, 8044, 8045, 8046, 8050, 8051, 8052, 8054, 8070, 8071, 8072, 8073, 8074, 8075, 8076, 8078, 8082, 8083, 8084, 8140, 8141, 8142, 8143, 8144, 8145, 8147, 8150, 8151, 8152, 8153, 8154, 8155, 8156, 8158, 8160, 8161, 8163, 8170, 8171, 8172, 8173, 8174, 8175, 8180, 8190, 8200, 8201, 8210, 8211, 8213, 8214, 8215, 8220, 8221, 8230, 8231, 8240, 8241, 8242, 8243, 8244, 8245, 8246, 8247, 8249, 8250, 8251, 8252, 8253, 8254, 8255, 8256, 8257, 8260, 8261, 8262, 8263, 8265, 8270, 8272, 8273, 8280, 8281, 8290, 8300, 8310, 8311, 8312, 8313, 8314, 8315, 8316, 8317, 8318, 8319, 8320, 8322, 8323, 8330, 8331, 8332, 8333, 8335, 8337, 8339, 8340, 8341, 8342, 8343, 8344, 8345, 8346, 8347, 8350, 8370, 8380, 8381, 8382, 8383, 8384, 8390, 8400, 8401, 8402, 8403, 8406, 8407, 8408, 8409, 8410, 8413, 8420, 8430, 8440, 8441, 8450, 8452, 8453, 8460, 8461, 8470, 8471, 8474, 8480, 8481, 8482, 8490, 8500, 8501, 8502, 8503, 8504, 8507, 8509, 8510, 8512, 8513, 8514, 8520, 8521, 8523, 8524, 8525, 8530, 8540, 8541, 8542, 8543, 8550, 8551, 8552, 8560, 8562, 8570, 8571, 8572, 8573, 8574, 8575, 8576, 8940, 8941, 8950, 8980, 8981, 8982, 8983, 9110

/3: grade poorly differentiated; /4: grade undifferentiated/anaplastic; /9: grade unknown; ICD: International Classification of Diseases.

Supplementary Table 2. Information on cancer stage at diagnosis available by cancer registry.

Cancer Registry	TNM stage	Other staging system
**Austria**[a,b]	Y	-
**Belgium**[a]	Y	-
**Bulgaria**[a]	Y	-
**Croatia**[a]	-	EOD
**Cyprus**[a]	-	EOD
**Czechia**[a]	Y	-
**Estonia**[a]	Y	EOD
**Germany**		
Baden-Württemberg	Y	-
Bavaria	Y	EOD
Brandenburg, Berlin	Y	-
Hamburg	Y	-
North Rhine-Westphalia	Y	-
Rhineland-Palatinate	Y	-
Schleswig-Holstein	Y	-
**Ireland**[a]	-	-
**Italy**		
Avellino	-	-
Basilicata	Y	-
Benevento	-	-
Bergamo	Y	-
Brescia	Y	-
Caserta	-	-
Catania, Messina, Enna	Y	-
Emilia-Romagna	-	-
Friuli Venezia Giulia	Y	-
Insubria-Como	Y	-
Insubria-Varese	Y	-
Lazio	-	-
Liguria	Y	EOD/FIGO/Dukes’
Mantova, Cremona	Y	-
Marche	Y	FIGO
Milan	Y	-
Montagna	Y	-
Monza-Brianza, Lecco	Y	-
Naples Centre	-	-
Napoli North	-	-
Napoli South	-	-
Nuoro	Y	EOD/Dukes’
Palermo	Y	-
Pavia	Y	-
Piedmont	-	-
Puglia	Y	EOD/Dukes’
Ragusa, Caltanissetta	Y	-
Reggio Calabria	Y	Dukes’
Salerno	-	-
Sassari	Y	EOD
Siracusa	Y	-
South Tyrol	Y	-
Trapani, Agrigento	Y	-
Trento	Y	-
Tuscany	-	-
Umbria	-	-
Valle d'Aosta	Y	-

Cancer Registry	TNM stage	Other staging system
Veneto	-	-
**Latvia[a]**	Y	FIGO
**Liechtenstein[a]**	Y	FIGO
**Lithuania[a]**	Y	-
**Netherlands[a]**	Y	EOD
**Portugal**		
Azores	-	-
**Romania**		
Cluj	Y	-
**Slovenia[a]**	Y	EOD/FIGO
**Spain**		
Asturias	-	-
Basque	Y	EOD
Canary Islands	-	-
Castilla Y Leon	Y	-
Castellón	Y	FIGO
Girona	Y	EOD/FIGO/Dukes'
Granada	-	-
La Rioja	Y	-
Murcia	Y	-
Navarra	-	-
Tarragona	Y	-
**Switzerland**		
Aargau	Y	-
Basel	Y	-
Bern Solothurn	Y	-
Fribourg	Y	-
Geneva	Y	-
Graubünden, Glarus	Y	-
Neuchâtel, Jura	Y	-
East Switzerland	Y	-
Ticino	Y	-
Valais	Y	-
Vaud	Y	-
Central Switzerland	Y	-
Zürich, Zug	Y	-
**UK**		
Northern Ireland	Y	-
Wales	Y	FIGO/Dukes'

Abbreviations: EOD: Extent of Disease; FIGO: International Federation of Gynecology and Obstetrics; TNM: Tumour-Nodes-Metastasis; Y: yes.
[a]National cancer registry; [b]Condensed TNM.

Supplementary Table 3. TNM edition adopted by cancer registry across calendar period.

Cancer Registry	TNM edition							
	1st	2nd	3rd	4th	5th	6th	7th	8th
**Austria**[a]	-	-	-	-	-	-	-	-
**Belgium**[a]	-	-	-	-	-	2004-2009	2010-2016	2017-2020
**Bulgaria**[a]	-	-	-	-	-	-	-	-
**Croatia**[a]	-	-	-	-	-	-	-	-
**Cyprus**[a]	-	-	-	-	-	-	-	-
**Czechia**[a]	-	-	1994-1994	1995-2000	2001-2004	2005-2010	2011-2018	-
**Estonia**[a]	-	-	-	-	-	-	1995-2019	2012-2019
**Germany**								
Baden-Württemberg	-	-	-	-	-	-	2015-2017	2018-2022
Bavaria					2003-2019	2003-2019	2003-2019	2003-2019
Brandenburg, Berlin	-	-	-	-	-	-	2017-2021	2017-2022
Hamburg	-	-	-	-	-	1995-2010	2010-2017	2017-2022
North Rhine-Westphalia	-	-	-	-	2010-2011	2010-2017	2010-2019	2010-2019
Rhineland-Palatinate	-	-	-	-	1998-2016	2002-2009	1999-2022	1998-2022
Schleswig-Holstein	-	-	-	-	1998-2003	2004-2017	2010-2020	2016-2020
**Ireland**[a]	-	-	-	-	-	-	-	-
**Italy**								
Avellino	-	-	-	-	-	-	-	-
Basilicata	-	-	-	-	-	2005-2008	2009-2017	2016-2018
Benevento	-	-	-	-	-	-	-	-
Bergamo	-	-	-	-	-	-	2007-2017	-
Brescia	-	-	-	-	-	-	-	-
Caserta	-	-	-	-	-	-	-	-
Catania, Messina, Enna	-	-	-	-	-	2003-2009	2010-2017	-
Emilia-Romagna	-	-	-	-	-	-	-	-
Insubria-Como	-	-	-	-	-	-	-	2003-2015
Insubria-Varese	-	-	-	-	-	-	-	1976-2015
Friuli Venezia Giulia	-	-	-	-	1995-2003	2004-2009	2010-2020	-
Lazio	-	-	-	-	-	-	-	-
Liguria	-	-	-	-	-	2011-2011	2011-2016	-
Mantova, Cremona	-	-	-	-	-	-	-	-
Marche	-	-	-	-	-	-	2010-2016	-
Milan	-	-	-	-	-	-	2013-2017	2013-2017
Montagna	-	-	-	-	-	-	-	-
Monza-Brianza, Lecco	-	-	-	-	-	-	2007-2015	-

Cancer Registry	TNM edition							
	1st	2nd	3rd	4th	5th	6th	7th	8th
Naples Centre	-	-	-	-	-	-	-	-
Naples North	-	-	-	-	-	-	-	-
Naples South	-	-	-	-	-	-	-	-
Nuoro	-	-	-	-	-	2003-2008	-	-
Palermo	-	-	-	-	-	-	2017-2017	-
Pavia	-	-	-	-	-	-	2016-2016	2003-2010
Piedmont	-	-	-	-	-	-	-	-
Puglia	-	-	-	-	-	-	2013-2018	2013-2018
Ragusa, Caltanissetta	-	-	-	-	-	-	1981-2012	2013-2017
Reggio Calabria	-	-	-	-	-	-	2010-2016	2017-2018
Salerno	-	-	-	-	-	-	-	-
Sassari	-	-	-	-	1992-2001	2002-2008	2009-2015	-
Siracusa	-	-	-	-	-	-	-	-
South Tyrol	-	-	-	-	-	-	1995-2017	-
Trapani, Agrigento	-	-	-	-	-	-	-	-
Trento	-	-	-	-	-	2009-2010	2011-2014	-
Tuscany	-	-	-	-	-	-	-	-
Umbria	-	-	-	-	-	-	-	-
Valle d'Aosta	-	-	-	-	-	-	2012-2016	-
Veneto	-	-	-	-	-	-	-	-
**Latvia**[a]	-	-	-	-	-	-	2014-2017	-
**Liechtenstein**[a]	-	-	-	-	-	-	2011-2016	2011-2021
**Lithuania**[a]	-	-	-	1991-2000	2001-2007	2008-2010	2011-2017	-
**Netherlands**[a]	-	-	-	1989-1998	1999-2002	2003-2009	2010-2016	2017-2020
**Portugal**								
Azores	-	-	-	-	-	-	-	-
**Romania**								
Cluj	-	-	-	-	-	-	2008-2016	-
**Slovenia**[a]	-	-	-	-	-	2003-2009	2010-2016	2017-2020
**Spain**								
Asturias	-	-	-	-	-	-	-	-
Basque	-	-	-	-	-	-	2010-2017	-
Canary Island	-	-	-	-	-	-	-	-
Castilla Y Leon	-	-	-	-	-	-	-	-
Castellon	-	-	-	-	-	2004-2009	2010-2017	2018-2018
Girona	-	-	-	1994-1997	1998-2002	2003-2009	2010-2018	2015-2020
Granada	-	-	-	-	-	-	-	-

Cancer Registry	TNM edition							
	1st	2nd	3rd	4th	5th	6th	7th	8th
La Rioja	-	-	-	-	-	1993-2009	2010-2017	-
Murcia	-	-	-	-	-	-	-	2016-2020
Navarra	-	-	-	-	-	-	-	-
Tarragona	-	-	-	-	2000-2002	2003-2009	2010-2017	2018-2018
**Switzerland**								
Aargau	-	-	-	-	-	-	2013-2016	2017-2019
Basel			1981-1985	1986-1997	1998-2002	2003-2009	2010-2016	2017-2019
Bern Solothurn	-	-	-	-	-	-	2014-2016	2014-2019
Fribourg	-	-	-	-	-	2006-2010	2010-2016	2017-2019
Geneva	1980-1986	1980-1983	1980-1991	1988-1998	1999-2002	2003-2013	2010-2018	2013-2019
Graubünden, Glarus	-	-	-	1989-1998	1999-2002	2003-2009	2010-2016	2017-2019
Neuchâtel, Jura	-	-	-	-	1980-2002	2003-2010	2011-2016	2017-2019
East Switzerland	1992-1998	1997-2002	1980-2005	1987-2004	1997-2002	1980-2010	1983-2016	1980-2019
Ticino	-	-	-	1996-1997	1998-2001	2002-2009	2010-2016	2017-2019
Valais	-	-	-	1989-1997	1998-2002	2003-2010	2010-2016	2017-2019
Vaud	-	-	-	-	1980-2002	2003-2010	2011-2017	2017-2017
Central Switzerland	-	-	-	-	-	-	2010-2017	2017-2019
Zürich, Zug	-	-	1980-1994	1995-1997	1998-2002	2003-2009	2007-2017	2013-2019
**UK**								
Northern Ireland	-	-	-	-	-	-	1993-2018	2018-2019
Wales	-	-	-	-	1995-2020	1995-2018	1996-2020	1997-2020

Abbreviation: TNM: Tumour-Nodes-Metastasis.
[a]National cancer registry.

Supplementary Table 4. Breast cancer cases with information on TNM stage by country across calendar periods.

Country		1990-92	1993-95	1996-98	1999-01	2002-04	2005-07	2008-10	2011-13	2014-16	2017-19	2020-22
**Austria[a]**	N	10614	12191	13523	13950	14366	14741	14744	15430	15269	15251	.
	%comp	95	93	91	92	90	89	89	91	89	86	.
	&unkn	0	0	0	0	0	0	0	0	0	0	.
	%miss	5	7	9	8	10	11	11	9	11	14	.
**Belgium[a]**	N	.	.	.	.	9621	29395	29905	32525	32343	33173	10696
	%comp	.	.	.	.	88	89	92	94	95	93	92
	&unkn	.	.	.	.	1	1	1	2	3	5	7
	%miss	.	.	.	.	11	10	6	4	2	2	1
**Bulgaria[a]**	N	.	7097	7646	8528	9824	10474	10858	11308	7364	.	.
	%comp	.	98	98	98	98	98	99	99	99	.	.
	&unkn	.	0	0	0	0	0	0	0	0	.	.
	%miss	.	2	2	2	2	2	1	1	1	.	.
**Croatia[a]**	N	.	.	.	1991	5886	6388	6744	7006	7604	7804	8026
	%comp	.	.	.	0	0	0	0	0	0	0	0
	&unkn	.	.	.	0	0	0	0	0	0	0	0
	%miss	.	.	.	100	100	100	100	100	100	100	100
**Cyprus[a]**	N	.	.	270	875	1101	1259	1356	1551	1749	1866	1990
	%comp	.	.	0	0	0	0	0	0	0	0	0
	&unkn	.	.	0	0	0	0	0	0	0	0	0
	%miss	.	.	100	100	100	100	100	100	100	100	100
**Czechia[a]**	N	.	7640	11848	13834	16088	17295	18135	19623	20368	13467	.
	%comp	.	95	92	93	93	96	98	98	96	95	.
	&unkn	.	0	0	0	0	0	0	0	0	0	.
	%miss	.	5	8	7	7	4	2	2	4	5	.
**Estonia[a]**	N	1244	1320	1552	1590	1765	1789	1913	2123	2204	2395	.
	%comp	0	25	96	97	97	96	92	92	93	93	.
	&unkn	0	0	0	0	0	0	0	0	1	0	.
	%miss	100	75	4	3	3	4	8	8	7	7	.
**Germany (7 registries)**	N	.	2444	12140	22868	41219	54501	75070	99860	116610	144659	63864
	%comp	.	57	78	89	90	90	87	84	82	82	86
	&unkn	.	24	11	4	4	3	4	6	6	7	5
	%miss	.	19	11	7	7	7	9	10	12	11	8
**Ireland[a]**	N	.	2914	4761	5511	6467	6931	8301	8708	9253	10420	6681
	%comp	.	0	0	0	0	0	0	0	0	0	0
	&unkn	.	0	0	0	0	0	0	0	0	0	0
	%miss	.	100	100	100	100	100	100	100	100	100	100
**Italy (38 registries)**	N	10901	15867	23214	30491	38173	46704	68338	95803	128776	70636	16997
	%comp	11	17	22	22	26	31	28	25	20	22	13
	&unkn	13	13	12	12	12	13	10	11	12	6	1
	%miss	77	70	66	66	62	56	61	63	68	73	86
**Latvia[a]**	N	.	.	.	1502	2357	2546	2604	2975	3113	1100	.
	%comp	.	.	.	91	86	91	94	98	99	98	.
	&unkn	.	.	.	1	1	2	2	1	0	0	.
	%miss	.	.	.	8	13	7	4	1	1	2	.
**Liechtenstein[a]**	N	.	.	.	.	.	.	.	96	76	107	45
	%comp	.	.	.	.	.	.	.	100	100	99	98
	&unkn	.	.	.	.	.	.	.	0	0	1	2
	%miss	.	.	.	.	.	.	.	0	0	0	0
**Lithuania[a]**	N	1428	2614	3089	3568	3495	3749	4033	4196	4391	1277	.
	%comp	94	99	98	99	99	98	97	97	98	98	.
	&unkn	0	0	0	0	0	0	0	0	0	0	.
	%miss	6	1	2	1	1	2	2	3	2	1	.
**Netherlands[a]**	N	26536	29498	30434	34353	35778	37749	40000	43093	44076	44832	13075
	%comp	97	97	98	99	99	99	99	98	96	94	93
	&unkn	2	2	2	1	1	1	1	2	4	6	7
	%miss	1	0	0	0	0	0	0	0	0	0	0
**Portugal - Azores**	N	.	.	182	269	287	282	367	368	387	.	.
	%comp	.	.	0	0	0	0	0	0	0	.	.
	&unkn	.	.	0	0	0	0	0	0	0	.	.
	%miss	.	.	100	100	100	100	100	100	100	.	.
**Romania - Cluj**	N	.	.	.	.	.	.	2076	2186	2331	.	.
	%comp	.	.	.	.	.	.	69	78	77	.	.

Country		1990-92	1993-95	1996-98	1999-01	2002-04	2005-07	2008-10	2011-13	2014-16	2017-19	2020-22
	&unkn	.	.	.	.	.	.	7	7	8	.	.
	%miss	.	.	.	.	.	.	24	15	14	.	.
**Slovenia[a]**	N	1960	2173	2614	2866	3205	3382	3555	3787	4075	4558	1444
	%comp	0	0	0	0	65	96	97	98	97	98	99
	&unkn	0	0	0	0	0	0	0	0	0	0	0
	%miss	100	100	100	100	35	4	3	2	2	2	1
**Spain**	N	5067	8285	9507	10694	11834	13239	15406	19406	19751	14935	1743
**(11 registries)**	%comp	1	5	8	14	18	20	28	40	46	76	95
	&unkn	0	1	1	2	1	1	2	3	3	4	2
	%miss	99	94	91	84	81	79	71	57	50	20	3
**Switzerland**	N	6202	6844	7990	8903	9087	9948	11131	13510	17402	17260	.
**(13 registries)**	%comp	89	88	88	91	95	96	96	96	95	92	.
	&unkn	6	10	10	8	4	3	3	3	4	7	.
	%miss	4	2	1	1	1	1	1	1	1	1	.
**UK - Northern**	N	.	2279	2506	2698	3045	3118	3467	3714	3921	4102	.
**Ireland**	%comp	.	54	81	81	86	86	92	93	96	98	.
	&unkn	.	0	0	0	0	0	0	0	2	1	.
	%miss	.	46	19	19	14	14	8	7	2	1	.
**UK - Wales**	N	.	1206	3316	4278	5444	6727	7322	7274	7924	7589	2092
	%comp	.	1	2	2	3	2	3	15	27	53	60
	&unkn	.	87	89	90	84	71	77	40	32	8	5
	%miss	.	12	9	8	14	27	20	45	41	39	35
**All countries**	N	63952	102372	134592	168769	219042	270217	325325	394542	448987	395431	126653
	%comp	69	67	66	67	69	71	69	67	63	70	66
	&unkn	4	5	6	6	5	5	5	6	7	5	4
	%miss	28	28	27	27	26	24	26	27	31	25	30

Abbreviations: N: total number of patients; TNM: Tumour-Nodes-Metastasis; %comp: percentage of patients with complete TNM stage information; %unkn: percentage of patients with unknown TNM stage information; %miss: percentage of patients with no TNM stage information.

[a]National cancer registry. Except for countries with a national coverage, the total number of cases may vary across calendar periods, depending on how many regional CRs contributed with data.

Supplementary Table 5. Cervical cancer cases with information on TNM stage by country across calendar periods.

Country		1990-92	1993-95	1996-98	1999-01	2002-04	2005-07	2008-10	2011-13	2014-16	2017-19	2020-22
**Austria[a]**	N	1991	1753	1573	1511	1462	1239	1140	1109	1019	957	.
	%comp	88	86	87	83	86	88	85	83	79	77	.
	&unkn	0	0	0	0	0	0	0	0	0	0	.
	%miss	12	14	13	17	14	12	15	17	21	23	.
**Belgium[a]**	N	.	.	.	.	625	1900	1824	1917	1937	1870	584
	%comp	.	.	.	.	64	68	75	79	82	90	92
	&unkn	.	.	.	.	2	3	4	3	5	5	3
	%miss	.	.	.	.	34	29	21	18	13	5	5
**Bulgaria[a]**	N	.	2264	2650	2909	3190	3365	3329	3198	2104	.	.
	%comp	.	96	98	97	97	98	95	94	93	.	.
	&unkn	.	0	0	0	1	1	2	1	1	.	.
	%miss	.	4	2	3	2	2	3	5	6	.	.
**Croatia[a]**	N	.	.	.	307	898	963	928	963	849	743	651
	%comp	.	.	.	0	0	0	0	0	0	0	0
	&unkn	.	.	.	0	0	0	0	0	0	0	0
	%miss	.	.	.	100	100	100	100	100	100	100	100
**Cyprus[a]**	N	.	.	27	58	57	72	86	100	105	115	102
	%comp	.	.	0	0	0	0	0	0	0	0	0
	&unkn	.	.	0	0	0	0	0	0	0	0	0
	%miss	.	.	100	100	100	100	100	100	100	100	100
**Czechia[a]**	N	.	2268	3126	3164	3097	2998	2985	2801	2466	1475	.
	%comp	.	85	80	84	88	92	95	95	93	90	.
	&unkn	.	0	0	0	0	0	0	0	0	0	.
	%miss	.	15	20	16	12	8	5	5	7	10	.
**Estonia[a]**	N	476	498	484	450	472	497	555	532	450	405	.
	%comp	0	0	0	0	0	0	0	63	93	93	.
	&unkn	0	0	0	0	0	0	0	0	1	1	.
	%miss	100	100	100	100	100	100	100	37	7	6	.
**Germany (7 registries)**	N	.	243	1099	1864	3043	3803	4712	6069	7150	8703	4049
	%comp	.	33	56	71	76	76	71	68	65	68	75
	&unkn	.	37	20	12	11	11	13	17	18	16	10
	%miss	.	30	24	17	14	14	16	14	17	16	15
**Ireland[a]**	N	.	324	550	520	604	772	951	919	816	863	496
	%comp	.	0	0	0	0	0	0	0	0	0	0
	&unkn	.	0	0	0	0	0	0	0	0	0	0
	%miss	.	100	100	100	100	100	100	100	100	100	100
**Italy (38 registries)**	N	931	1327	1624	1796	2097	2448	3395	4585	5879	2840	601
	%comp	2	9	18	16	19	20	16	13	9	8	1
	&unkn	8	10	10	9	10	10	9	7	7	3	1
	%miss	90	82	72	75	71	70	75	79	84	89	98
**Latvia[a]**	N	.	.	.	339	600	628	608	696	717	192	.
	%comp	.	.	.	79	74	80	77	86	86	80	.
	&unkn	.	.	.	0	1	2	3	2	0	1	.
	%miss	.	.	.	21	25	18	20	12	14	20	.
**Liechtenstein[a]**	N	.	.	.	.	.	.	.	4	2	2	1
	%comp	.	.	.	.	.	.	.	0	0	0	0
	&unkn	.	.	.	.	.	.	.	0	0	0	0
	%miss	.	.	.	.	.	.	.	100	100	100	100
**Lithuania[a]**	N	577	969	1214	1348	1364	1360	1377	1181	967	295	.
	%comp	90	97	97	98	97	97	95	93	92	93	.
	&unkn	0	0	0	0	0	0	1	0	0	0	.
	%miss	10	3	3	2	3	3	5	7	8	7	.
**Netherlands[a]**	N	2268	2184	2208	2011	1967	2120	2155	2119	2247	2516	803
	%comp	96	97	97	97	98	98	97	97	98	99	98
	&unkn	3	2	2	2	2	2	2	3	2	1	1
	%miss	1	1	1	0	1	0	0	0	0	0	0
**Portugal - Azores**	N	.	.	24	41	56	51	44	36	57	.	.
	%comp	.	.	0	0	0	0	0	0	0	.	.
	&unkn	.	.	0	0	0	0	0	0	0	.	.
	%miss	.	.	100	100	100	100	100	100	100	.	.
**Romania - Cluj**	N	.	.	.	.	.	.	1255	1239	1087	.	.
	%comp	.	.	.	.	.	.	14	28	23	.	.

Country		1990-92	1993-95	1996-98	1999-01	2002-04	2005-07	2008-10	2011-13	2014-16	2017-19	2020-22
	&unkn	.	.	.	.	.	.	13	46	58	.	.
	%miss	.	.	.	.	.	.	73	27	19	.	.
**Slovenia**[a]	N	510	553	650	608	593	495	400	381	352	293	131
	%comp	98	97	98	98	98	98	98	97	96	96	98
	&unkn	0	0	0	0	0	0	0	0	0	0	0
	%miss	2	3	2	2	2	2	3	3	4	4	2
**Spain**	N	628	896	949	974	1029	1200	1276	1399	1351	1020	110
**(11 registries)**	%comp	0	1	4	8	10	14	15	15	20	44	84
	&unkn	0	0	0	1	1	2	1	2	2	3	7
	%miss	100	99	96	91	90	83	84	83	78	53	9
**Switzerland**	N	549	584	600	517	460	432	476	549	700	669	.
**(13 registries)**	%comp	43	40	46	41	63	75	72	77	79	83	.
	&unkn	9	17	23	14	13	12	17	10	12	12	.
	%miss	48	42	30	45	24	13	12	13	9	5	.
**UK - Northern**	N	.	234	248	235	223	273	326	295	239	245	.
**Ireland**	%comp	.	59	81	88	91	91	94	99	99	99	.
	&unkn	.	0	0	0	0	0	0	0	0	1	.
	%miss	.	41	19	12	9	9	6	1	0	0	.
**UK - Wales**	N	.	115	340	420	453	493	473	443	475	467	124
	%comp	.	2	0	7	12	16	52	75	89	89	97
	&unkn	.	6	2	8	14	18	3	4	1	1	.
	%miss	.	92	98	84	74	67	45	21	10	10	3
**All countries**	N	7930	14212	17366	19072	22290	25109	28295	30535	30969	23670	7652
	%comp	66	69	68	68	68	68	63	62	58	64	61
	&unkn	2	3	3	3	3	4	5	7	8	7	6
	%miss	32	29	28	29	29	28	32	31	33	29	33

Abbreviations: N: total number of patients; TNM: Tumour-Nodes-Metastasis; %comp: percentage of patients with complete TNM stage information; %unkn: percentage of patients with unknown TNM stage information; %miss: percentage of patients with no TNM stage information.

[a]National cancer registry. Except for countries with a national coverage, the total number of cases may vary across calendar periods, depending on how many regional CRs contributed with data.

Supplementary Table 6. Colorectal cancer cases with information on TNM stage by country across calendar periods.

Country		1990-92	1993-95	1996-98	1999-01	2002-04	2005-07	2008-10	2011-13	2014-16	2017-19	2020-22
**Austria[a]**	N	11659	12500	13595	13606	13890	13693	13050	12327	12006	11315	.
	%comp	91	91	90	90	89	88	88	89	88	86	.
	&unkn	0	0	0	0	0	0	0	0	0	0	.
	%miss	9	9	10	10	11	12	12	11	12	14	.
**Belgium[a]**	N	.	.	.	.	7362	22517	23764	24447	25454	22606	6813
	%comp	.	.	.	.	83	86	89	92	94	95	97
	&unkn	.	.	.	.	1	2	2	2	2	3	2
	%miss	.	.	.	.	15	12	8	6	3	2	1
**Bulgaria[a]**	N	.	5103	5999	7396	9183	11496	12390	12162	8232	.	.
	%comp	.	95	98	98	99	99	99	98	97	.	.
	&unkn	.	0	0	0	0	0	0	0	0	.	.
	%miss	.	4	2	2	1	1	1	2	3	.	.
**Croatia[a]**	N	.	.	.	2203	6698	7308	8125	8342	8853	8054	7827
	%comp	.	.	.	0	0	0	0	0	0	0	0
	&unkn	.	.	.	0	0	0	0	0	0	0	0
	%miss	.	.	.	100	100	100	100	100	100	100	100
**Cyprus[a]**	N	.	.	153	568	660	833	921	1027	1092	1083	1192
	%comp	.	.	0	0	0	0	0	0	0	0	0
	&unkn	.	.	0	0	0	0	0	0	0	0	0
	%miss	.	.	100	100	100	100	100	100	100	100	100
**Czechia[a]**	N	.	10212	17544	20037	21877	21686	22838	22376	22254	13528	.
	%comp	.	89	87	87	88	92	96	94	92	90	.
	&unkn		0	0	0	0	0	0	0	0	0	.
	%miss	.	11	13	13	12	8	4	6	8	10	.
**Estonia[a]**	N	1238	1385	1601	1759	1921	2013	2089	2439	2534	2709	.
	%comp	0	25	91	94	94	92	88	90	92	92	.
	&unkn	0	0	0	0	0	0	0	1	2	1	.
	%miss	100	75	9	6	6	8	12	9	6	7	.
**Germany (7 registries)**	N	.	1862	10377	20104	39269	50214	61209	79420	90179	110495	42780
	%comp	.	47	71	82	86	84	82	81	80	81	85
	&unkn	.	28	10	4	4	5	5	7	6	7	5
	%miss	.	25	19	14	11	11	13	12	13	12	10
**Ireland[a]**	N	.	3100	4737	4968	5481	6133	6647	6877	7189	7306	4507
	%comp	.	0	0	0	0	0	0	0	0	0	0
	&unkn	.	0	0	0	0	0	0	0	0	0	0
	%miss	.	100	100	100	100	100	100	100	100	100	100
**Italy (38 registries)**	N	9570	15307	21588	26898	34688	45647	61722	79845	100108	49734	10274
	%comp	13	15	15	13	26	32	30	26	20	22	13
	&unkn	7	6	6	6	7	9	8	9	9	4	0
	%miss	80	78	80	81	67	59	63	66	71	74	86
**Latvia[a]**	N	.	.	.	1448	2318	2433	2320	2776	2685	924	.
	%comp	.	.	.	95	93	93	92	95	93	92	.
	&unkn	.	.	.	0	1	2	3	1	0	0	.
	%miss	.	.	.	5	6	5	6	4	7	8	.
**Liechtenstein[a]**	N	.	.	.	.	.	.	.	50	44	63	40
	%comp	.	.	.	.	.	.	.	100	98	98	98
	&unkn	.	.	.	.	.	.	.	0	0	2	0
	%miss	.	.	.	.	.	.	.	0	2	0	3
**Lithuania[a]**	N	1203	2216	2825	3395	3539	3591	3944	4053	3464	1229	.
	%comp	83	94	95	95	96	96	94	91	92	92	.
	&unkn	0	0	0	0	0	0	0	0	0	0	.
	%miss	17	6	5	5	4	4	6	9	8	8	.
**Netherlands[a]**	N	21906	23327	24916	26672	29588	33141	36445	38612	45732	40149	10923
	%comp	95	95	95	96	96	96	95	96	96	97	97
	&unkn	4	4	4	3	3	4	4	4	4	3	2
	%miss	1	1	1	1	1	0	0	0	0	0	0
**Portugal - Azores**	N	.	.	111	208	237	249	278	309	369	.	.
	%comp	.	.	0	0	0	0	0	0	0	.	.
	&unkn	.	.	0	0	0	0	0	0	0	.	.
	%miss	.	.	100	100	100	100	100	100	100	.	.
**Romania - Cluj**	N	.	.	.	.	.	.	2085	2432	2506	.	.
	%comp	.	.	.	.	.	.	72	70	68	.	.

Country		1990-92	1993-95	1996-98	1999-01	2002-04	2005-07	2008-10	2011-13	2014-16	2017-19	2020-22
	&unkn	.	.	.	.	.	.	6	4	4	.	.
	%miss	.	.	.	.	.	.	22	26	28	.	.
**Slovenia[a]**	N	2010	2370	2727	3035	3540	3825	4610	4376	4052	3868	1224
	%comp	0	0	0	0	61	90	93	95	95	96	97
	&unkn	0	0	0	0	0	0	0	0	0	0	1
	%miss	100	100	100	100	39	10	7	5	5	4	2
**Spain**	N	5053	8456	10134	11881	14098	16221	19445	24225	23166	15798	1429
**(11 registries)**	%comp	0	0	1	6	14	18	29	41	45	72	93
	&unkn	0	1	1	2	1	1	1	2	4	4	3
	%miss	100	99	98	91	85	81	70	56	52	24	4
**Switzerland**	N	5117	5384	6067	6351	6645	7404	7658	8978	11740	11004	.
**(13 registries)**	%comp	61	70	72	79	94	94	94	93	93	93	.
	&unkn	6	9	10	10	4	4	4	4	4	5	.
	%miss	34	21	18	11	2	2	2	3	3	1	.
**UK - Northern**	N	.	2295	2417	2378	2505	2795	2946	3240	2966	3052	.
**Ireland**	%comp	.	68	75	77	87	88	92	94	95	94	.
	&unkn	.	0	0	0	0	0	1	0	2	5	.
	%miss	.	32	25	22	13	12	8	6	2	1	.
**UK - Wales**	N	.	819	2841	4178	4903	5516	6208	6058	6003	6135	1741
	%comp	.	0	2	3	2	3	3	12	30	67	68
	&unkn	.	17	9	35	49	60	66	44	32	5	4
	%miss	.	83	89	63	49	38	32	45	38	28	29
**All countries**	N	57756	94336	127632	157085	208402	256715	298694	344371	380628	309052	88750
	%comp	64	62	61	62	66	68	67	65	63	71	66
	&unkn	3	3	3	4	4	5	5	5	5	4	3
	%miss	33	35	35	35	30	27	28	29	32	24	31

Abbreviations: N: total number of patients; TNM: Tumour-Nodes-Metastasis; %comp: percentage of patients with complete TNM stage information; %unkn: percentage of patients with unknown TNM stage information; %miss: percentage of patients with no TNM stage information.

[a]National cancer registry. Except for countries with a national coverage, the total number of cases may vary across calendar periods, depending on how many regional CRs contributed with data.

Supplementary Table 7. Gastric cancer cases with information on TNM stage by country across calendar periods.

Country		1990-92	1993-95	1996-98	1999-01	2002-04	2005-07	2008-10	2011-13	2014-16	2017-19	2020-22
**Austria**[a]	N	5131	4920	4806	4245	3977	3805	3424	3360	2990	2872	.
	%comp	87	84	84	83	80	76	75	75	75	74	.
	&unkn	0	0	0	0	0	0	0	0	0	0	.
	%miss	13	16	16	17	20	24	25	25	25	26	.
**Belgium**[a]	N	.	.	.	.	1296	3859	3749	3851	3524	3491	1062
	%comp	.	.	.	.	68	69	73	80	83	87	89
	%Un	.	.	.	.	4	2	4	5	6	6	6
	%miss	.	.	.	.	29	29	22	15	11	7	6
**Bulgaria**[a]	N	.	2167	2352	2436	3072	3747	3897	3571	2234	.	.
	%comp	.	97	98	97	97	96	97	96	94	.	.
	&unkn	.	0	0	0	0	1	0	1	0	.	.
	%miss	.	3	2	2	3	3	3	4	6	.	.
**Croatia**[a]	N	.	.	.	899	2469	2403	2204	2374	2049	1775	1593
	%comp	.	.	.	0	0	0	0	0	0	0	0
	&unkn	.	.	.	0	0	0	0	0	0	0	0
	%miss	.	.	.	100	100	100	100	100	100	100	100
**Cyprus**[a]	N	.	.	52	152	162	178	229	221	218	219	198
	%comp	.	.	0	0	0	0	0	0	0	0	0
	&unkn	.	.	0	0	0	0	0	0	0	0	0
	%miss	.	.	100	100	100	100	100	100	100	100	100
**Czechia**[a]	N	.	3239	4720	4597	4543	4335	4342	4148	3766	2446	.
	%comp	.	82	78	77	78	80	92	91	90	87	.
	&unkn	.	0	0	0	0	0	0	0	0	0	.
	%miss	.	18	22	23	22	20	8	9	10	13	.
**Estonia**[a]	N	1259	1276	1314	1198	1112	1169	1094	1031	1030	985	.
	%comp	0	0	0	0	0	0	0	56	86	88	.
	&unkn	0	0	0	0	0	0	0	1	1	1	.
	%miss	100	100	100	100	100	100	100	43	13	12	.
**Germany (7 registries)**	N	.	549	2998	5175	9039	11901	14369	18519	20847	25006	10293
	%comp	.	47	59	69	71	69	60	58	58	63	71
	&unkn	.	20	10	5	5	6	8	13	11	13	10
	%miss	.	33	31	25	24	25	32	29	30	24	19
**Ireland**[a]	N	.	826	1258	1222	1210	1300	1437	1482	1561	1369	891
	%comp	.	0	0	0	0	0	0	0	0	0	0
	&unkn	.	0	0	0	0	0	0	0	0	0	0
	%miss	.	100	100	100	100	100	100	100	100	100	100
**Italy (38 registries)**	N	5376	7625	9686	11303	12276	14192	18487	23410	28256	13719	2696
	%comp	8	11	10	9	13	16	15	14	12	9	5
	&unkn	2	2	3	3	4	5	4	4	3	2	0
	%miss	89	88	87	88	83	78	80	82	85	89	95
**Latvia**[a]	N	.	.	.	978	1423	1490	1252	1226	1259	393	.
	%comp	.	.	.	95	94	92	90	91	90	89	.
	&unkn	.	.	.	1	1	2	2	1	0	0	.
	%miss	.	.	.	5	5	6	8	8	10	11	.
**Liechtenstein**[a]	N	.	.	.	.	.	.	.	14	9	14	8
	%comp	.	.	.	.	.	.	.	100	89	79	88
	&unkn	.	.	.	.	.	.	.	0	11	21	13
	%miss	0	0	0	0	0	0	0	0	0	0	0
**Lithuania**[a]	N	1168	2057	2454	2555	2432	2215	2132	2136	1669	534	.
	%comp	79	93	93	93	94	93	85	83	80	81	.
	&unkn	0	0	0	0	0	0	0	0	0	0	
	%miss	21	7	7	7	6	7	15	17	20	19	.
**Netherlands**[a]	N	7308	6796	6325	5980	5762	5420	5542	5293	4783	4741	1576
	%comp	82	83	80	82	83	84	84	87	89	90	92
	&unkn	17	16	18	18	16	15	15	13	11	10	7
	%miss	1	1	1	1	1	0	0	0	0	0	0
**Portugal - Azores**	N	.	.	105	130	155	130	135	145	164	.	.
	%comp	.	.	0	0	0	0	0	0	0	.	.
	&unkn	.	.	0	0	0	0	0	0	0	.	.
	%miss	.	.	100	100	100	100	100	100	100	.	.
**Romania - Cluj**	N	.	.	.	.	.	.	1076	1073	998	.	.
	%comp	.	.	.	.	.	.	55	58	53	.	.

Country		1990-92	1993-95	1996-98	1999-01	2002-04	2005-07	2008-10	2011-13	2014-16	2017-19	2020-22
	&unkn	.	.	.	.	.	.	3	3	4	.	.
	%miss	.	.	.	.	.	.	42	39	43	.	.
**Slovenia[a]**	N	1201	1274	1306	1288	1295	1296	1317	1313	1251	1175	372
	%comp	0	0	0	0	48	69	75	81	82	83	87
	&unkn	0	0	0	0	2	3	3	2	2	2	3
	%miss	100	100	100	100	49	28	22	18	16	15	10
**Spain (11 registries)**	N	2865	3599	3617	3795	3662	3946	4301	4951	4472	2960	292
	%comp	0	0	0	2	5	11	12	13	15	20	34
	&unkn	0	0	0	3	3	1	3	2	1	2	5
	%miss	100	100	99	95	92	88	85	85	84	78	61
**Switzerland (13 registries)**	N	1553	1493	1613	1400	1331	1380	1472	1779	2282	2224	.
	%comp	45	54	58	50	67	76	71	70	75	79	.
	&unkn	11	13	15	13	13	11	9	10	10	12	.
	%miss	44	33	27	37	20	14	20	20	15	9	.
**UK - Northern Ireland**	N	.	592	659	658	643	597	614	588	565	503	.
	%comp	.	0	2	21	5	19	46	69	76	87	.
	&unkn	.	0	0	0	0	0	0	0	14	12	.
	%miss	.	100	98	79	95	81	54	31	10	0	.
**UK - Wales**	N	.	299	822	1246	1319	1227	1161	1133	1088	1032	257
	%comp	.	1	1	1	0	1	1	2	7	18	14
	&unkn	.	1	1	10	18	16	16	12	7	2	2
	%miss	.	98	99	89	82	83	83	86	86	80	84
**All countries**	N	25861	36712	44087	49257	57178	64590	72234	81618	85015	65458	19238
	%comp	48	50	48	47	50	52	49	48	45	52	54
	&unkn	6	4	4	4	4	4	5	6	5	7	7
	%miss	46	46	48	49	45	43	46	47	50	41	40

Abbreviations: N: total number of patients; TNM: Tumour-Nodes-Metastasis; %comp: percentage of patients with complete TNM stage information; %unkn: percentage of patients with unknown TNM stage information; %miss: percentage of patients with no TNM stage information.

[a]National cancer registry. Except for countries with a national coverage, the total number of cases may vary across calendar periods, depending on how many regional CRs contributed with data.

Supplementary Table 8. Lung cancer cases with information on TNM stage by country across calendar periods.

Country		1990-92	1993-95	1996-98	1999-01	2002-04	2005-07	2008-10	2011-13	2014-16	2017-19	2020-22
**Austria[a]**	N	7658	8220	9073	9723	10305	10624	11026	11140	11718	11127	.
	%comp	88	88	87	86	85	84	83	84	85	80	.
	&unkn	0	0	0	0	0	0	0	0	0	0	.
	%miss	12	12	13	14	15	16	17	16	15	20	.
**Belgium[a]**	N	.	.	.	.	6696	20452	21478	22586	22991	23741	7803
	%comp	.	.	.	.	69	68	78	84	90	95	95
	&unkn	.	.	.	.	2	2	2	1	2	1	1
	%miss	.	.	.	.	29	31	21	14	8	4	3
**Bulgaria[a]**	N	.	4126	4433	5125	5895	7553	8880	8876	5400	.	.
	%comp	.	92	97	98	97	98	98	97	95	.	.
	&unkn	.	0	0	0	0	0	0	0	0	.	.
	%miss	.	8	3	2	3	2	2	3	5	.	.
**Croatia[a]**	N	.	.	.	2541	7069	6322	5653	6479	6654	7294	7453
	%comp	.	.	.	0	0	0	0	0	0	0	0
	&unkn	.	.	.	0	0	0	0	0	0	0	0
	%miss	.	.	.	100	100	100	100	100	100	100	100
**Cyprus[a]**	N	.	.	98	397	458	499	677	825	887	1025	1243
	%comp	.	.	0	0	0	0	0	0	0	0	0
	&unkn	.	.	0	0	0	0	0	0	0	0	0
	%miss	.	.	100	100	100	100	100	100	100	100	100
**Czechia[a]**	N	.	8121	12987	13961	14326	14935	15747	15873	15899	10332	.
	%comp	.	86	84	82	83	83	91	93	93	91	.
	&unkn	.	0	0	0	0	0	0	0	0	0	.
	%miss	.	14	16	18	17	17	9	7	7	9	.
**Estonia[a]**	N	1624	1661	1607	1687	1661	1630	1668	1850	1983	1982	.
	%comp	0	0	0	0	0	0	0	64	94	94	.
	&unkn	0	0	0	0	0	0	0	0	1	0	.
	%miss	100	100	100	100	100	100	100	35	5	6	.
**Germany (7 registries)**	N	.	1529	7810	13333	22930	30099	42300	61864	75610	97831	42683
	%comp	.	45	56	69	73	71	69	68	74	79	86
	&unkn	.	19	9	5	4	4	5	7	6	7	5
	%miss	.	36	34	26	23	25	26	25	20	14	10
**Ireland[a]**	N	.	2239	3372	3472	4018	4512	5282	5943	6316	6735	4206
	%comp	.	0	0	0	0	0	0	0	0	0	0
	&unkn	.	0	0	0	0	0	0	0	0	0	0
	%miss	.	100	100	100	100	100	100	100	100	100	100
**Italy (38 registries)**	N	9530	13127	17102	19649	23514	29004	41411	58874	76803	39892	8281
	%comp	4	6	8	7	10	12	10	10	9	7	3
	&unkn	2	2	2	3	4	5	3	5	6	3	0
	%miss	94	92	90	90	86	83	87	85	84	90	97
**Latvia[a]**	N	.	.	.	990	1666	2075	2076	2231	2091	758	.
	%comp	.	.	.	90	86	85	79	86	92	93	.
	&unkn	.	.	.	1	2	3	4	1	0	0	.
	%miss	.	.	.	9	12	12	16	13	8	7	.
**Liechtenstein[a]**	N	.	.	.	.	.	.	.	55	54	58	24
	%comp	.	.	.	.	.	.	.	98	98	97	92
	&unkn	.	.	.	.	.	.	.	2	0	0	0
	%miss	.	.	.	.	.	.	.	0	2	3	8
**Lithuania[a]**	N	1397	2663	3179	3477	3245	2913	2853	2686	2261	857	.
	%comp	83	95	96	94	96	94	90	88	88	86	.
	&unkn	0	0	0	0	0	0	0	0	0	0	.
	%miss	17	5	4	6	4	6	10	12	12	14	.
**Netherlands[a]**	N	24947	25206	25255	24859	26463	28842	31392	32790	33786	35084	11381
	%comp	88	90	91	94	95	97	97	98	98	99	99
	&unkn	9	8	7	5	4	2	2	2	1	1	1
	%miss	3	2	2	2	2	1	1	0	0	0	0
**Portugal - Azores**	N	.	.	161	245	267	272	329	352	388	.	.
	%comp	.	.	0	0	0	0	0	0	0	.	.
	&unkn	.	.	0	0	0	0	0	0	0	.	.
	%miss	.	.	100	100	100	100	100	100	100	.	.
**Romania - Cluj**	N	.	.	.	.	.	.	1659	1750	1711	.	.
	%comp	.	.	.	.	.	.	71	69	65	.	.

Country		1990-92	1993-95	1996-98	1999-01	2002-04	2005-07	2008-10	2011-13	2014-16	2017-19	2020-22
	&unkn	.	.	.	.	.	.	3	3	3	.	.
	%miss	.	.	.	.	.	.	26	28	32	.	.
**Slovenia[a]**	N	2470	2642	2741	2899	3139	3323	3367	3493	3952	4272	1355
	%comp	0	0	0	0	55	82	88	95	96	97	99
	&unkn	0	0	0	0	3	4	2	1	1	0	0
	%miss	100	100	100	100	42	15	10	4	3	3	1
**Spain (11 registries)**	N	4603	7145	8029	8869	9796	11203	12983	15899	16048	12374	1373
	%comp	0	1	2	8	12	17	24	38	43	62	48
	&unkn	0	0	0	1	1	1	1	1	1	1	0
	%miss	100	99	98	91	87	83	76	61	55	37	51
**Switzerland (13 registries)**	N	4780	4856	5416	5625	5782	6704	7210	8730	11604	11808	.
	%comp	40	46	48	52	70	76	76	85	88	90	.
	&unkn	11	12	13	10	12	10	8	6	4	5	.
	%miss	49	43	39	38	18	14	16	10	8	6	.
**UK - Northern Ireland**	N	.	1635	1827	1718	1651	1998	2259	2457	2668	2693	.
	%comp	.	13	23	24	24	76	86	92	98	98	.
	&unkn	.	0	0	0	0	0	0	0	1	2	.
	%miss	.	87	77	76	76	24	14	8	1	0	.
**UK - Wales**	N	.	880	2393	3165	3452	4529	5159	5058	5253	5107	1433
	%comp	.	0	0	1	1	0	1	2	9	17	15
	&unkn	.	0	1	4	8	9	13	7	8	2	1
	%miss	.	100	99	95	92	91	87	91	83	81	84
**All countries**	N	57009	84050	105483	121735	152333	187489	223409	269811	304077	272970	87235
	%comp	56	56	55	55	57	59	59	58	58	68	66
	&unkn	5	4	3	3	3	3	3	3	4	4	3
	%miss	38	40	41	42	40	38	38	38	38	29	31

Abbreviations: N: total number of patients; TNM: Tumour-Nodes-Metastasis; %comp: percentage of patients with complete TNM stage information; %unkn: percentage of patients with unknown TNM stage information; %miss: percentage of patients with no TNM stage information.

[a]National cancer registry. Except for countries with a national coverage, the total number of cases may vary across calendar periods, depending on how many regional CRs contributed with data.

Supplementary Table 9. Prostate cancer cases with information on TNM stage by country across calendar periods.

Country		1990-92	1993-95	1996-98	1999-01	2002-04	2005-07	2008-10	2011-13	2014-16	2017-19	2020-22
**Austria[a]**	N	6120	8620	11689	14815	16480	15564	13989	12611	13227	15547	.
	%comp	87	82	80	80	82	81	78	80	81	77	.
	&unkn	0	0	0	0	0	0	0	0	0	0	.
	%miss	13	18	20	20	18	19	22	20	19	23	.
**Belgium[a]**	N	.	.	.	.	9677	27885	26239	25195	25206	29266	9575
	%comp	.	.	.	.	63	64	73	82	84	93	96
	&unkn	.	.	.	.	2	2	2	2	3	1	0
	%miss	.	.	.	.	36	34	25	16	13	6	4
**Bulgaria[a]**	N	.	1290	1751	2467	3514	4040	4743	8124	4657	.	.
	%comp	.	95	96	98	97	97	98	98	97	.	.
	&unkn	.	0	0	0	0	1	0	0	0	.	.
	%miss	.	5	4	2	3	2	2	2	3	.	.
**Croatia[a]**	N	.	.	.	949	3170	3935	4566	4595	5715	7141	5509
	%comp	.	.	.	0	0	0	0	0	0	0	0
	&unkn	.	.	.	0	0	0	0	0	0	0	0
	%miss	.	.	.	100	100	100	100	100	100	100	100
**Cyprus[a]**	N	.	.	139	611	780	910	1130	1329	1352	1445	2078
	%comp	.	.	0	0	0	0	0	0	0	0	0
	&unkn	.	.	0	0	0	0	0	0	0	0	0
	%miss	.	.	100	100	100	100	100	100	100	100	100
**Czechia[a]**	N	.	3900	7249	8180	11030	14530	18060	20502	20832	15484	.
	%comp	.	61	67	68	75	87	96	96	95	91	.
	&unkn	.	0	0	0	0	0	0	0	0	0	.
	%miss	.	39	33	32	25	13	4	4	5	9	.
**Estonia[a]**	N	473	742	882	968	1564	2229	2849	3369	3316	3283	.
	%comp	0	0	0	0	0	0	0	56	89	91	.
	&unkn	0	0	0	0	0	0	0	3	3	3	.
	%miss	100	100	100	100	100	100	100	41	8	6	.
**Germany**	N	.	1149	6762	16247	39073	51514	63924	83635	90726	127377	58439
**(7 registries)**	%comp	.	35	53	62	65	64	58	55	56	65	73
	&unkn	.	30	19	14	14	14	18	24	22	18	13
	%miss	.	35	29	24	21	22	24	20	23	17	13
**Ireland[a]**	N	.	1915	3118	4328	6236	7269	8720	9906	9627	11197	7382
	%comp	.	0	0	0	0	0	0	0	0	0	0
	&unkn	.	0	0	0	0	0	0	0	0	0	0
	%miss	.	100	100	100	100	100	100	100	100	100	100
**Italy**	N	3774	7817	13527	20403	31242	37542	51358	66387	81383	46233	11045
**(38 registries)**	%comp	2	2	3	3	4	5	6	8	8	7	1
	&unkn	3	7	9	7	8	9	7	7	7	3	2
	%miss	94	91	88	90	88	87	87	85	85	90	98
**Latvia[a]**	N	.	.	.	839	1819	2282	2297	2543	3119	1207	.
	%comp	.	.	.	76	72	78	83	93	95	98	.
	&unkn	.	.	.	2	3	5	6	3	0	0	.
	%miss	.	.	.	22	26	17	11	4	5	2	.
**Liechtenstein[a]**	N	.	.	.	.	.	.	.	66	57	84	72
	%comp	.	.	.	.	.	.	.	98	100	96	99
	&unkn	.	.	.	.	.	.	.	2	0	0	1
	%miss	.	.	.	.	.	.	.	0	0	4	.
**Lithuania[a]**	N	396	921	1571	2604	4937	9613	10150	9141	8454	2168	.
	%comp	84	96	96	94	92	87	79	72	69	75	.
	&unkn	0	0	0	0	0	0	0	0	0	0	.
	%miss	16	4	4	6	8	13	21	28	31	25	.
**Netherlands[a]**	N	13451	18109	19600	20549	24857	27988	30431	33478	31548	36712	11942
	%comp	86	90	93	97	98	97	97	99	99	99	100
	&unkn	12	8	6	1	1	2	3	0	0	0	0
	%miss	2	2	1	1	1	1	1	0	0	0	0
**Portugal -**	N	.	.	105	266	298	324	399	343	311	.	.
**Azores**	%comp	.	.	0	0	0	0	0	0	0	.	.
	&unkn	.	.	0	0	0	0	0	0	0	.	.
	%miss	.	.	100	100	100	100	100	100	100	.	.
**Romania - Cluj**	N	.	.	.	.	.	.	1209	1098	1099	.	.
	%comp	.	.	.	.	.	.	27	31	35	.	.

Country		1990-92	1993-95	1996-98	1999-01	2002-04	2005-07	2008-10	2011-13	2014-16	2017-19	2020-22
	&unkn	.	.	.	.	.	.	15	18	17	.	.
	%miss	.	.	.	.	.	.	58	51	48	.	.
**Slovenia[a]**	N	659	819	1220	1636	2119	2866	4002	4636	4806	4871	1415
	%comp	0	0	0	0	36	56	52	47	50	54	71
	&unkn	0	0	0	0	15	27	30	32	30	28	13
	%miss	100	100	100	100	49	17	19	21	19	18	16
**Spain**	N	2116	4223	6569	9529	12404	14523	16729	19754	18638	14922	1479
**(11 registries)**	%comp	0	0	1	2	6	13	13	13	16	22	45
	&unkn	0	0	1	3	4	2	3	3	2	3	1
	%miss	100	100	99	95	90	84	84	84	82	74	54
**Switzerland**	N	4870	5761	7018	8124	9459	10371	11486	13071	16722	18942	.
**(13 registries)**	%comp	40	39	42	64	69	78	82	77	80	85	.
	&unkn	17	20	18	13	19	12	8	6	11	14	.
	%miss	43	41	40	23	12	10	10	16	9	1	.
**UK - Northern**	N	.	1029	1021	1273	1888	2340	2723	2780	2999	3242	.
**Ireland**	%comp	.	0	2	5	6	8	71	98	98	97	.
	&unkn	.	0	0	0	0	0	0	0	1	2	.
	%miss	.	100	98	95	94	91	29	2	1	1	.
**UK - Wales**	N	.	636	2001	3621	5270	6230	6675	6387	6888	7901	1868
	%comp	.	0	0	0	0	0	0	4	4	7	6
	&unkn	.	0	0	2	6	4	8	7	9	12	9
	%miss	.	100	99	98	93	95	92	88	87	81	86
**All countries**	N	31859	56931	84222	117409	185817	241955	281679	328950	350682	347022	110804
	%comp	60	54	51	50	52	54	52	52	51	60	59
	&unkn	8	6	6	5	6	6	7	9	9	9	8
	%miss	32	40	43	45	42	40	41	39	40	31	33

Abbreviations: N: total number of patients; TNM: Tumour-Nodes-Metastasis; %comp: percentage of patients with complete TNM stage information; %unkn: percentage of patients with unknown TNM stage information; %miss: percentage of patients with no TNM stage information.

[a]National cancer registry. Except for countries with a national coverage, the total number of cases may vary across calendar periods, depending on how many regional CRs contributed with data.

Supplementary Figure 1. General European population-based cancer registries providing information on cancer stage at diagnosis, September 2025.

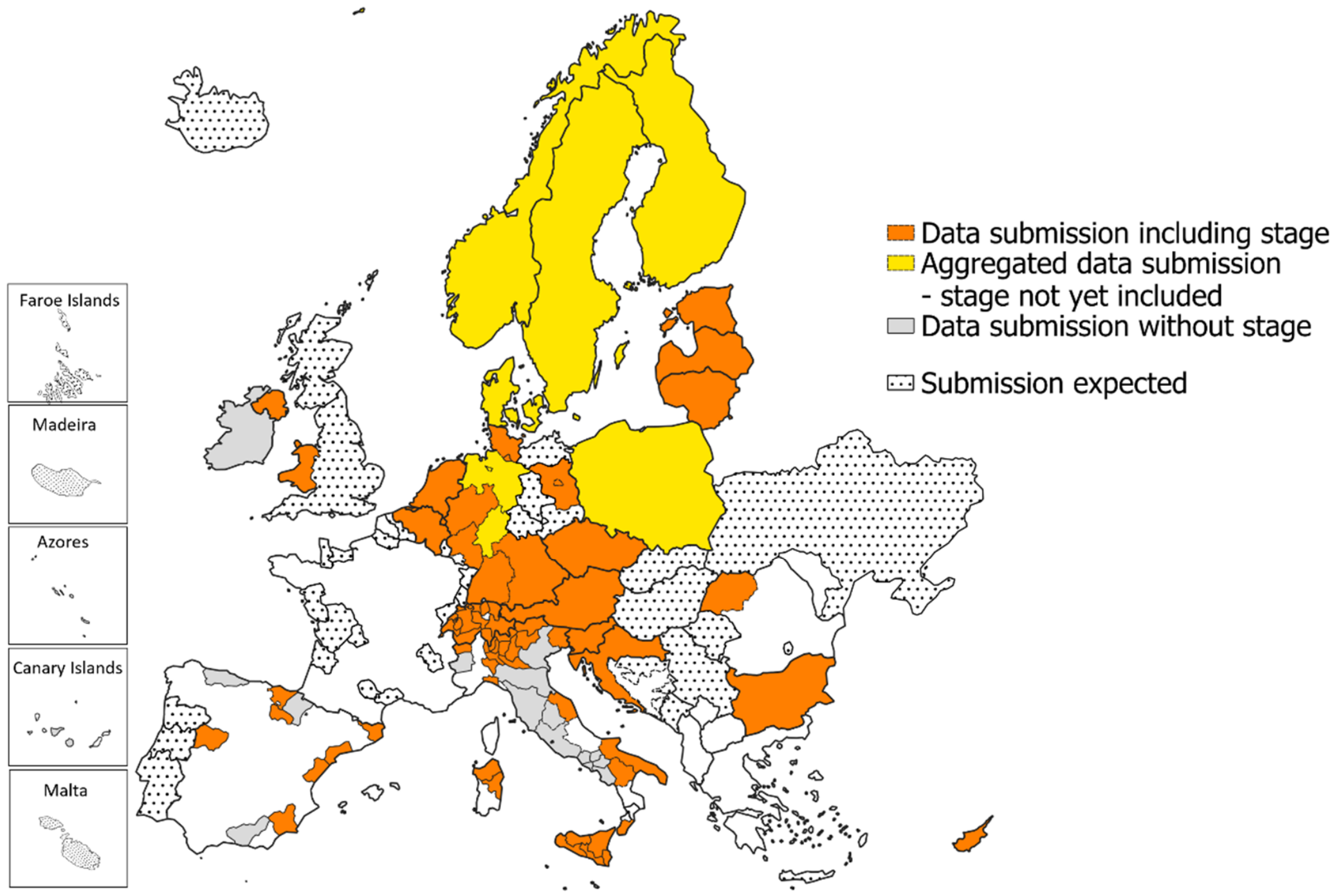

Supplement: Supplementary file 1 [file IJC-159-2540-s001.pdf]
